# Membrane lipid remodeling eradicates *Helicobacter pylori* by manipulating the cholesteryl 6'-acylglucoside biosynthesis

**DOI:** 10.1186/s12929-024-01031-8

**Published:** 2024-04-29

**Authors:** Lih-Lih Ong, Hau-Ming Jan, Hong-Hanh Thi Le, Tsai-Chen Yang, Chou-Yu Kuo, Ai-Feng Feng, Kwok-Kong Tony Mong, Chun-Hung Lin

**Affiliations:** 1 Institute of Biological Chemistry, Academia Sinica, No. 128, Academia Road Section 2, Nan-Kang, Taipei, 11529 Taiwan; 2 Department of Applied Chemistry, National Yang Ming Chiao Tung University, 1001, University Road, Eastern District, Hsinchu, 300093 Taiwan; 3 Institute of Chemistry, Academia Sinica, No. 128, Academia Road Section 2, Nan-Kang, Taipei, 11529 Taiwan; 4 Sustainable Chemical Science and Technology, Taiwan International Graduate Program, Academia Sinica, No. 128, Academia Road Section 2, Nan-Kang, Taipei, 11529 Taiwan; 5 Joint Program in Transfusion Medicine, Department of Pathology, Brigham and Women’s Hospital, Harvard Medical School, Boston, USA; 6 Department of Chemistry and Institute of Biochemical Sciences, National Taiwan University, Taipei, 10617 Taiwan

**Keywords:** Adhesion, Biosynthesis, Cholesterol, *Helicobacter pylori*, Membrane, Phospholipids

## Abstract

**Background:**

*Helicobacter pylori*, the main cause of various gastric diseases, infects approximately half of the human population. This pathogen is auxotrophic for cholesterol which it converts to various cholesteryl α-glucoside derivatives, including cholesteryl 6’-acyl α-glucoside (CAG). Since the related biosynthetic enzymes can be translocated to the host cells, the acyl chain of CAG likely comes from its precursor phosphatidylethanolamine (PE) in the host membranes. This work aims at examining how the acyl chain of CAG and PE inhibits the membrane functions, especially bacterial adhesion.

**Methods:**

Eleven CAGs that differ in acyl chains were used to study the membrane properties of human gastric adenocarcinoma cells (AGS cells), including lipid rafts clustering (monitored by immunofluorescence with confocal microscopy) and lateral membrane fluidity (by the fluorescence recovery after photobleaching). Cell-based and mouse models were employed to study the degree of bacterial adhesion, the analyses of which were conducted by using flow cytometry and immunofluorescence staining, respectively. The lipidomes of *H. pylori,* AGS cells and *H. pylori*–AGS co-cultures were analyzed by Ultraperformance Liquid Chromatography-Tandem Mass Spectroscopy (UPLC-MS/MS) to examine the effect of PE(10:0)_2_, PE(18:0)_2_, PE(18:3)_2_, or PE(22:6)_2_ treatments.

**Results:**

CAG10:0, CAG18:3 and CAG22:6 were found to cause the most adverse effect on the bacterial adhesion. Further LC–MS analysis indicated that the treatment of PE(10:0)_2_ resulted in dual effects to inhibit the bacterial adhesion, including the generation of CAG10:0 and significant changes in the membrane compositions. The initial (1 h) lipidome changes involved in the incorporation of 10:0 acyl chains into dihydro- and phytosphingosine derivatives and ceramides. In contrast, after 16 h, glycerophospholipids displayed obvious increase in their very long chain fatty acids, monounsaturated and polyunsaturated fatty acids that are considered to enhance membrane fluidity.

**Conclusions:**

The PE(10:0)_2_ treatment significantly reduced bacterial adhesion in both AGS cells and mouse models. Our approach of membrane remodeling has thus shown great promise as a new anti-*H. pylori* therapy.

**Graphical Abstract:**

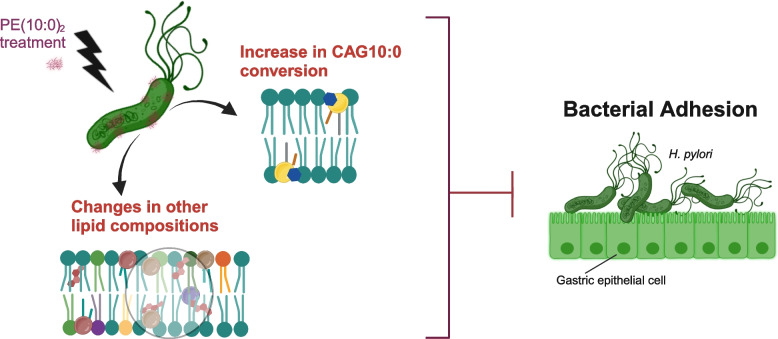

**Supplementary Information:**

The online version contains supplementary material available at 10.1186/s12929-024-01031-8.

## Background

Infecting more than one half of the world population, *Helicobacter pylori* represents the main cause of various gastric diseases, including gastritis, gastric ulcer, duodenum ulcer and gastric carcinoma [1, 2]. This pathogen was categorized as Group I carcinogen by World Health Organization (WHO) in 1994 [3]. Emergence of multiple antibiotic resistance is the primary challenge in current therapeutic treatments, explaining the importance to develop a new type of antibiotics. Colonization in the stomach by *H. pylori* is the major risk of gastric cancer [4]. Prior to colonization, the bacterium adheres to gastric epithelium cells, which is the first and vital step for the pathogenesis [4, 5]. So far, several adhesins were shown to contribute to the bacterial adherence to host cells, such as BabA/B [6–8] and SabA [4, 9]. Additionally, cholesterol-derived metabolites also play a crucial role in the adhesion of *H. pylori* [10, 11]; especially this bacterium is auxotrophic for cholesterol and has to hijack cholesterol from the host cells [12]. Upon uptake, the bacterial enzyme cholesteryl α-glucosyltransferase (CGT, encoded by *hp0421*) catalyzes the formation of cholesteryl α-glucoside (CG) [13]. CG is subjected to further transformation; an acyl or phosphatidyl transfer occurs at 6’-hydroxyl group of CG, leading to the formation of cholesteryl 6’-acyl or 6’-phosphatidyl α-glucoside (namely CAG or CPG), respectively [14]. Cholesteryl α-glucoside 6’-acyltransferase (CGAT, encoded by *hp0499*) is the enzyme responsible for the acyl transfer from phosphatidylethanolamine (PE) to CG, leading to the formation of CAG. CGAT was previously identified and characterized in depth for its role in the bacterial pathogenesis [15, 16].

Furthermore, ΔCGT, a CGT-deletion mutant lack of any cholesteryl glucoside derivative, was shown to display impaired adhesion to epithelial cells [10]. We recently pinpointed CAG, but not CG or CPG, to enhance lipid raft clustering that is essential for the adhesion of *H. pylori* [15]. This bacterium was shown to secrete outer membrane vesicles (OMVs) that contain the necessary enzymes for CAG biosynthesis [17]. Since the OMVs can be delivered and fused with the host cell membranes, the encompassed enzymes, including CGT and CGAT, can thus directly access host lipids (such as cholesterol and PE) to produce CAGs. The resulting CAGs therefore contain different acyl chains, considering that PEs in animals usually consist of several saturated and unsaturated acyl chains such as palmitic acid (designated as 16:0), stearic acid (18:0), oleic acid (18:1), linoleic acid (18:2), arachidonic acid (20:4) and docosahexaenoic acid (22:6). We herein report how the chain length and unsaturation of CAGs influence membrane features, such as lipid rafts clustering, membrane fluidity and rigidity. Intriguingly, CAGs that have the 10:0-, 18:3- and 22:6-acyl chains showed the most negative effect on the bacterial adhesion. The result inspired us to develop a therapeutic approach for eradication of *H. pylori*, i.e., direct addition of the biosynthetic precursor PE to gastric epithelial cells or mice. Further mass spectrometric analysis pinpointed how the PE(10:0)_2_ treatment became the best because it produced a significant amount of CAG10:0 in vivo, and that it also caused significant changes in the membrane compositions. Additional mice study demonstrated that the PE(10:0)_2_-containing diet was able to effectively prevent the bacterial adhesion.

## Methods

### Preparation of CAGs

The CAGs investigated in this work were synthesized according to the published procedure [18]. These compounds were characterized in details to confirm their structures, as shown by ^1^H and ^13^C NMR spectra (that are shown as appendixes after Supplemental Fig. S10).

### *H. pylori* strains and bacterial culture

*H. pylori* 26695 strains, were sourced from the American Type Culture Collection (ATCC), was grown on CDC Anaerobic Blood Agar plates (Becton Dickinson) under microaerobic (Anaaeropack Campylo System, Mitsubishi Gas Chemical, Tokyo, Japan) conditions at 37 °C for 2 days. For liquid cultures, *H. pylori w*as grown in Brucella broth (Difco) containing 0.2% β-cyclodextrin (Sigma), 10% fetal bovine serum, and 1% IsoVitaleX (Becton Dickinson) for 60 h. Clinical isolated MDR strain MDR4955 was obtained from National Taiwan University Hospital, Taiwan. It is resistant to clarithromycin, metronidazole, and levofloxacin and their resistance breakpoints are defined as > 0.5, > 8, and > 1 µg ml^−1^, respectively.

### Cell lines, cell culture and immunofluorescent staining

AGS (human gastric epithelial, female) cells were obtained from the American Type Culture Collection (ATCC) and were certified by ATCC. ATCC uses morphology-, karyotyping-, and PCR-based approaches to confirm the identity of this cell line. AGS cells were tested negative for mycoplasma using PCR-based approach. AGS cells were maintained within a humidified environment under 5% CO_2_ at 37 ℃ in Dulbecco’s modification of Eagle’s medium (abbreviated as DMEM; Gibco, Invitrogen) supplemented with fetal bovine serum (10%; Hyclone) and penicillin/streptomycin (1%; Biological Industries).

AGS cells were allowed to grow up to 80% confluence in 12-well plates prior to the addition of PE or CAG. For staining of lipid rafts, AGS cells were prepared as aforementioned and treated with an indicated CAG for 1 h. Alexa Fluor 594-conjugated cholera toxin subunit b (CT-b) was utilized to label lipid rafts (GM1) in AGS cells, followed by fluorescence microscopy to visualize the clustering of GM1. For immunofluorescent staining, cells were fixed with 2% formaldehyde and stained with *H. pylori*-specific antibody (Abcam; 1:500 dilution) at 4 °C. Nuclei were counterstained with DAPI (blue). Goat anti-rabbit FITC-conjugated secondary antibody (Invitrogen, 1:1000) was applied to the cells at 4 °C for 1 h, and the free antibodies were washed away with PBS. Immunofluorescence microscopy was performed. Each channel's z-stack was projected onto a single plane using the maximum z-projection function in ImageJ software, followed by the merging of all channels.

### Measurement of Fluorescence recovery after photobleaching (FRAP)

AGS cells were seeded on a 35-mm round-glass coverslip and treated with an indicated CAG (at a final concentration of 20 µM), followed by the labeling with Alexa Fluor 594-conjugated CT-b in phenol red-free DMEM medium (Gibco, Invitrogen) at 4 °C for 30 min. Cells were washed with PBS and mounted in the pre-warmed Hanks’ balanced salt solution (supplemented with 25 mM HEPES, pH 7.4) on a Zeiss LSM 700 confocal laser-scanning microscope equipped with a stage heater at 37 °C. FRAP analysis was performed with a × 63 NA 1.4 Plan-Apochromat oil-immersion objective. For quantitative analysis, pre- and post-photobleaching images were monitored with 488 nm laser line of a 10-mW diode laser set to 40% laser power at 2.0% transmission and with a maximum confocal pinehole set. Photobleaching of a circular region of interest (ROI) with a diameter of 3.0 µm was performed by 60 bleach iterations with the diode laser set to 100% transmission. Cell images were taken at 3 s intervals for 2 min. Fluorescence intensities of the bleached ROI, the whole cell and background fluorescence were recorded by using ZEN 2009 software package (Carl Zeiss, Jena, Germany). To analyze the recovered fluorescence intensities in the ROI region, the signal intensities were corrected by background signal (*F*_*B*_). [*F*(*t*)_*FRAP-NORM*_] was collected by multiplying the ratio of post-bleached ROI intensity [*F*(*t*)_*ROI*_] to the whole cell intensity [*F*(*t*)_*WC*_], with the ratio of pre-bleaching intensities (*F*_*Pre*-*ROI*_* / F*_*Pre*_) according to the equation described as:

1 $${F(t)}_{FRAP-NORM}={[(F(t)}_{ROI}- {F}_{B})/{(F(t)}_{WC}-{F}_{B})]{[(F(t)}_{Pre}-{F}_{B})/{(F(t)}_{Pre-ROI}-{F}_{B})]$$

The curve of fluorescence recovery was plotted over time and fitted to the one phase association equation, *I*(*t*) = *A*(1–*e*^-*kt*^), where *k* is the rate constant. The half-life of recovery (*t*_1/2_) was calculated as *t*_1/2_ = ln 2/*k* described by Phair et al. [19]. Mobile fractions (*M*_*f*_) were calculated in accordance with Eq. 2.

2 $${M}_{f}={[(F}_{\mu }-{F}_{0})/{(F}_{Pre}-{F}_{0})]$$

${F}_{\mu },{F}_{0}$ and ${F}_{Pre}$ are designated as the normalized fluorescence intensities at *t* = 290, *t* = 0, and the time before the bleach, respectively.

### Co-culture experiments with CAG or PE treatment

Co-culture experiments of AGS and *H. pylori* cells were performed as previously described in Jan et al. [17]. For membrane dynamics studies, AGS cells (5 × 10^5^) were seeded in a 35-mm tissue-culture dish containing DMEM (Gibco, Invitrogen) at 37 °C for 16 h. Medium was replaced with serum-free Ham’s F-12 medium before the infection study. In experiments involving only cells, the *H. pylori* 26695 cells or AGS cells were subjected to treatment with CAG or PE for an indicated period of time. For infection experiments, the AGS cells were infected with *H. pylori* 26695 (with or without CAG or PE treatment) at a multiplicity of infection (MOI) of 50:1 for 1 h. After PBS washes for three times, cells were collected for the imaging with confocal microscopy or mass spectrometric (MS) analysis.

For the bacterial adhesion assay, AGS cells were seeded at 0.75 × 10^6^ confluency in a 6 mm tissue culture dish containing DMEM (Gibco, Invitrogen) at 37 °C for 12 h, and refreshed with serum-free Ham’s F-12 medium before bacterial infection. *H. pylori* 26695 (or the MDR strain MDR4955) was first treated with an indicated CAG or PE (Avanti Polar Lipids) and then co-cultured with AGS cells (MOI = 50). The non-adherent bacteria were washed away upon harvest. Adherence was measured by flow cytometry analysis as the proportion of adhered cells with *H. pylori*.

### Flow cytometry analysis

Cells treated with CAG were infected with *H. pylori* 26695 (MOI = 50) at 37 °C for 1 h. After the cells were washed to remove non-adherent bacteria, they were detached with 2 mM EDTA and fixed with 2% formaldehyde. Cells with plasma membrane-associated *H. pylori* were stained with rabbit anti-*H. pylori* antibody (Abcam, ab20459, 1:1000) for 16 h at 4 °C and then washed with PBS. The secondary antibody, FITC-conjugated goat anti-rabbit antibody (Invitrogen, 1:1000), was applied at 4 °C for 1 h and the resulting samples were washed with PBS. Cells were then analyzed by a flow cytometry system (BD FACSCalibur Calibur Flow Cytometer 4 Color) which is coupled with BD FACStation Software (Version 3.3) for data acquisition and analysis. Bacterial adherence was quantitated in terms of the proportion of cells with adherent *H. pylori*. At least 10,000 cells were collected.

### Sample preparation and subsequent lipid and metabolite analysis

*H. pylori* 26695 was incubated with 50 µM 17β-([3’-Azidopropoxy)-5-androsten-3β-ol (an azide-containing analogue of cholesterol) under microaerobic conditions at 37 °C for 16 h and then treated with PE(10:0)_2_, PE(18:3)_2_ or PE(22:6)_2_. The bacterial cells were collected, mixed with 0.9% KCl and subjected to Folch partitioning [20], in which 2-step extraction was performed using the mixture of chloroform:methanol (1:2 and 2:1; v/v). The organic layer was evaporated to give a dried residue that was redissolved in chloroform/methanol/water (5:4:1, v/v/v) and subjected to a click reaction with 0.25 mM alkyne-dye (4-N-methylamino-1,8- napthalimidopropyne, abbreviated as MAN) in the presence of 1.25 mM tris (benzyltriazolylmethyl)amine, 12.5 mM sodium ascorbate, and 0.25 mM CuSO_4_ at room temperature for 1 h. After the samples were dried, they were stored at -20 °C until HPLC or MS analysis.

Ultrahigh-performance liquid chromatography (UPLC; Waters)-MS (Orbitrap Elite, Thermo), equipped with an analytical CSH C18 column (1.7 mm, 100 × 2.1 mm; Waters, USA), was applied for the quantitative analysis of CAGs (Supplemental Fig. S5). CAGs were chromatographed at a flow rate of 0.4 mL min^−1^ with a gradient from a mixture of acetonitrile/water (30/70, v/v) with 20 mM ammonium acetate to a mixture of isopropyl alcohol/acetonitrile (90/10, v/v) with 20 mM ammonium acetate. The analysis of other lipids was conducted by untargeted LC–MS and the mass experiments were performed with positive electrospray ion mode set to one full FT-MS scan (m/z 200–1600, 15,000 resolution) and switched to different FT-MS product ion scans (15,000 resolution) for different precursors of cholesteryl glucoside derivatives. Both collision-induced dissociation and higher energy collisional dissociation were utilized to perform the fragmentation. For sensitive quantitation, multiple reaction monitoring was performed, which detected the MAN fragment at 366.16 m/z., with details regarding transitions and source conditions for each metabolite according to previous procedure [15, 17, 18]. MS raw data files were processed by MS-Dial software (v.5.1.23) [21]. The processes include smoothing, peak deconvolution, compound identification, and alignment. Metabolite annotation was performed using in-house mzRT libraries using the Metabolomics Standards Initiative (MSI) with first level of identification, matching only m/z and retention time (RT).

### Mouse treated and infected to measure the degree of bacterial infection

This study was approved by the Institutional Animal Care and Use Committee, Academia Sinica. All experimental tests were conducted in accordance with the regulations listed by the Guide for the Care and Use of Laboratory Animals. Seven-week-old C57BL/6 (B6) male mice, free of specific pathogens (including *Helicobacter* spp.), were purchased from BioLASCO Taiwan Co., Ltd., and housed at the Infectious Disease Core Facility in Biomedical Translation Research Center, Academia Sinica. *H. pylori* 26695 was grown under microaerobic conditions (Anaeropack Campylo System, Mitsubishi Gas Chemical, Tokyo, Japan) at 37 °C for 2 days on CDC Anaerobic Blood Agar plates (Becton Dickinson). The mice were starved for one day before they were treated with PE(10:0)_2_ and *H. pylori* infection for 2 phases. At day 1 of the first and second phases, the mice were treated with 1000 mg/kg per mouse of PE(10:0)_2_ (purchased from AMBINTER, at a dose of 1000 mg/kg in 200 μl of Brucella broth) by intragastrically. At days 2, 3 and 4 of the two phases, the bacteria (10^10^ cells in 200 μl of Brucella broth) and PE(10:0)_2_ (at a dose of 1000 mg/kg) were administered to the mice by intragastrically (see Fig. 6). The experiment was in accordance with modified procedure [22, 23]. The mice were euthanized four weeks post-the infection. The stomachs were halved longitudinally and rinsed in sterile DPBS. Half of the stomach was homogenized using a mechanical homogenizer in Brucella broth containing 10% FBS at room temperature. The appropriate volume of serial dilutions was plated on Brucella agar supplemented with 10% FBS, *H. pylori-*selective supplement (Dent Oxoid™; SR0147), and 2.5 international unit (IU) ml^−1^ polymyxin B to determine the degree of colonization (CFU). The other half of the stomach was formalin-fixed, embedded in optimum cutting temperature compound and sectioned to perform immunofluorescence staining for *H. pylori*.

### Immunohistochemistry

The other half of the stomach were immediately fixed with 4% formaldehyde for 24 h after the dissection, The formaldehyde-fixed tissues were soaked overnight in 30% sucrose fixative solution. Prior to tissue sectioning, the sucrose-treated tissues were embedded into optimum cutting temperature compound (OCT). The tissue was sectioned at a thickness of 10 μm at − 20 °C, and followed by 0.1% (v/v) Triton X-100 permeabilization in PBS for 30 min. The cryo-section was applied for antibodies staining after it was blocked with blocking reagent (5% normal goat serum and 0.3% Triton TX-100 in PBS) for 1 hour. The nuclei of murine gastric epithelial cells were shown in blue (by using Hoechst 33342 dye). The bacteria were labeled in green (by using *H. pylori*-specific monoclonal antibody and 488-conjugated secondary antibody). All images were acquired by confocal laser microscopy.

### Isolation of outer membrane vesicles (OMVs) and subsequent immunoblotting analysis

OMVs were purified were isolated by using a modified previous method [24–26]. The cells of *H. pylori* 26695 were cultured in 20 ml of Brucella broth (Difco) containing 0.2% β-cyclodextrin (Sigma), 10% fetal bovine serum, and 1% IsoVitaleX (Becton Dickinson) with initial OD_600_ of 0.05, incubated at 37 °C under microaerobic conditions. The resulting culture media were harvested at specified time intervals (24 h, 48 h, and 72 h), followed by centrifugation at 4,000 rpm at 4 °C for 20 min. The supernatant was filtered through a 0.45 μm filter and subsequently subjected to ultracentrifugation at 40,000 rpm at 4 °C for 2 h. The resulting pellet was washed three times with DPBS (Dulbecco’s phosphate-buffered saline), with each wash followed by centrifugation. OMVs were resuspended with 200 µl DPBS and then the protein and lipid content were determined by the Bradford assay and lipophilic styryl dye, N-(3-triethylammoniumpropyl)-4-(6-(4-(diethylamino) phenyl) hexatrienyl) pyridinium dibromide (FM4-64; Santa Cruz Biotechnology) using an Infinite M1000 PRO microtiter plate reader (Tecan Group Ltd., Männedorf, Switzerland). The analysis of OMVs (30 µg protein) was conducted by Western blot, in which total proteins were labeled by using the No-Stain™ protein labeling reagent (Thermo-Fisher Scientific). The rabbit anti-VacA polyclonal antibodies (HPP-5013–9, Austral Biologicals, San Ramon, CA, USA) with a 1:2000 dilution and peroxidase (HRP)-conjugated polyclonal goat anti-rabbit antibodies (1:3000; Boster Biological Technology) were used to detect the VacA protein.

### Statistics and reproducibility

The results are expressed as mean ± SD (standard deviation). For the graphs, data were combined from at least three biological independent experiments. Statistical significance between two sample groups was tested by an unpaired t-test using Prism 8.0 software (GraphPad Software, La Jolla, CA). All statistically significant differences are indicated with asterisks; ****p < 0.0001, ***p < 0.001, **p < 0.01, *p < 0.05 (not significant, *p* > 0.05). Statistical comparison was performed with unpaired Student's t-test or one-way ANOVA between control group and treatment groups. The statistical significance of the fold change of each lipid class was calculated, and then False-Discovery Rate (FDR) was calculated to pick up statistically significant metabolites with *p*-value < 0.05, FDR < 0.01. MetaboAnalyst 5.0 (https://www.metaboanalyst.ca) [27] to assess the variability within the sample group, employing techniques such as PCA and Heat map. Each sample's lipid peak areas were normalized by dividing them by the total peak area of all lipids. Circular dendrograph was plotted using graph with R version 4.3.0 (https://www.r-project.org).

## Results

### CAG’s acyl chain affects lipid rafts clustering in gastric epithelium

**Fig. 1:**
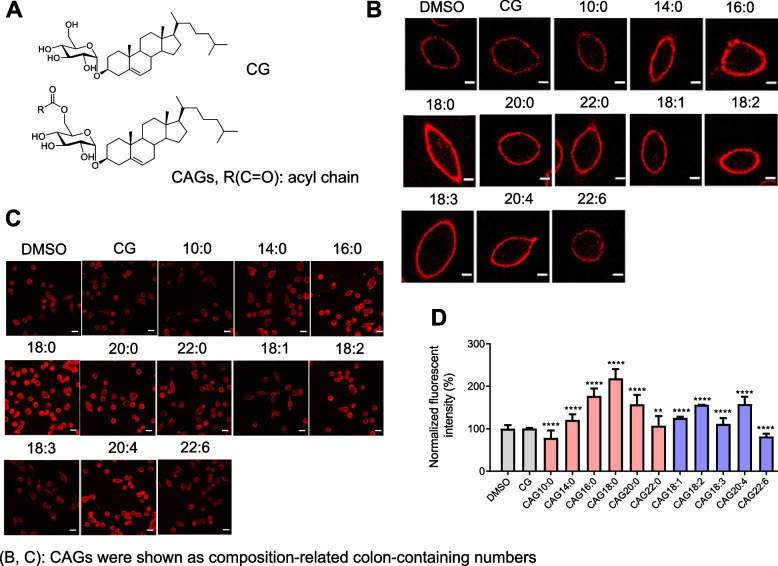
Treatment of AGS cells with CAGs and CG resulted in a different degree of lipids rafts clustering.** A** Structures of CAGs and CG in which R(C = O) denotes an acyl chain. **B** and **C** Confocal images of lipid rafts clustering in the presence of CAGs and CG in a single (**B**) or multiple AGS cells (**C**). After AGS cells were treated with CAGs or CG (as indicated) for I h, lipid rafts (GM1-containing) were labeled with Alexa Fluor 594-conjugated cholera toxin subunit b (CT-b; red fluorescence). Confocal images were collected under a Leica SP5 X inverted confocal microscope. Scale bar: 5 μm. Please note that colon-containing numbers are designated as CAGs that contain corresponding acyl chains. **D** The fluorescent intensities in (**C**) were quantified. At least 40 GM1-positive AGS cells from each experiment were scored for quantitative analysis by using ImageJ software. Statistical analysis was performed using Student's *t* test, and the asterisks represent statistical significance (*P* < 0.05) compared with the DMSO control group. (*p < 0.05, **p < 0.01, ***p < 0.001, ****p < 0.0001)

CAG was shown to enhance lipid rafts clustering in the host cell membranes, which is beneficial for the adherence of *H. pylori* to host cells [15]. The key biosynthetic enzyme CGAT displays broad specificity for donor substrates, including different types of phospholipids (e.g., PE, phosphatidylserine (PS) and phosphatidylcholine (PC)) and phospholipids of various acyl chains [17]. We therefore prepared a series of CAGs and analogues with various saturated or unsaturated acyl moieties attached (Fig. 1A). After AGS cells (a human gastric adenocarcinoma cell line) were individually treated with CAGs or analogues for 1 h, the degree of lipid rafts clustering was monitored by confocal microscopy in which lipid rafts were labeled by Alexa Fluor 594-conjugated cholera toxin subunit b (CT-b, red fluorescence) [28], as shown in Fig. 1B, C. The degree of lipid rafts clustering in Fig. 1C was quantified (see Fig. 1D). Representative three-dimensional cell images upon CAG treatments were shown in Movie S1. Interestingly, the GM1-fluorescence intensity became higher with a longer chain length when AGS cells were treated with CAG10:0, CAG14:0, CAG16:0 or CAG18:0, but an opposite trend was observed when AGS cells were treated with CAG18:0, CAG20:0 or CAG22:0. Obviously, the treatment with CAG18:0 led to the highest level of lipid rafts clustering, likely due to its best stacking with membrane lipids. Furthermore, we also examined the CAGs containing unsaturated acyl chains, they were less efficient to promote lipid rafts clustering than their saturated counterparts. The result of CAG10:0 and CAG22:6 particularly drew our attention because they are the only two to reduce the level of lipid rafts clustering, as compared to that in the negative control (treatment with DMSO).

### Effect of an acyl chain in CAG on lateral membrane fluidity

**Fig. 2:**
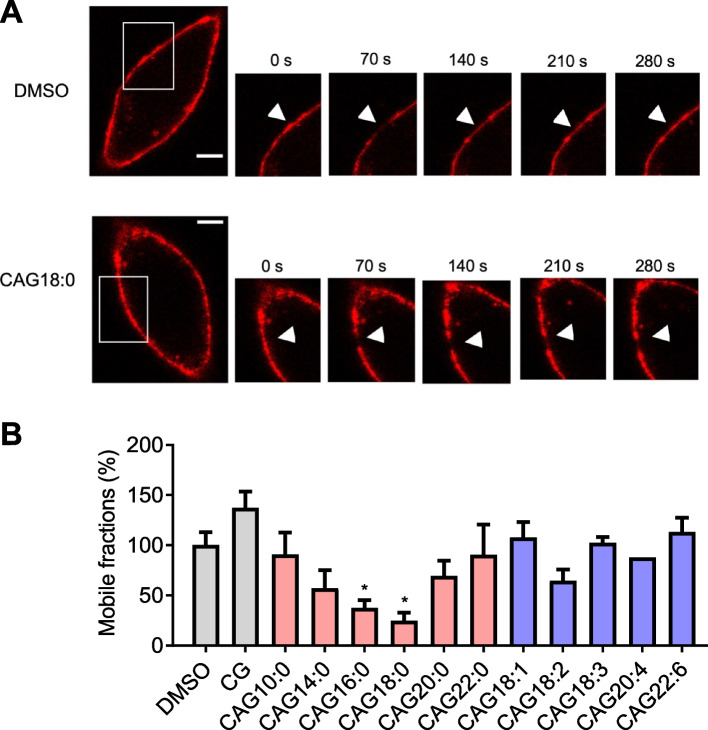
Acyl chains of CAGs affected membrane fluidity in AGS cells. **A** Representative images were collected from the fluorescence recovery after photobleaching (FRAP). AGS cells were treated with different CAGs or DMSO (control) and labeled with Alexa Fluor 594-conjugated CT-b (red fluorescence). A region of interest with a 3.0 μm diameter (arrow) was photobleached with intense lesser pulses at 595 nm and the recovery of fluorescence was recorded at 20 s intervals. Scale Bar: 5 mm. **B** Comparisons of the mobile fractions (*M*_*f*_) for CAGs and CG. ${M}_{f}$ is calculated by ${[(F}_{\mu }- {F}_{0})/ {(F}_{Pre}- {F}_{0})]$. ${F}_{\mu }, {F}_{0} \hspace{1 mm} {{\text{and}} \hspace{1 mm} F}_{Pre}$ are designated as post-bleach steady state, initial post-bleach, and pre-bleach fluorescent intensities, respectively. The values are means and standard deviations of five independent experiments. Statistical analysis employed the Student's t-test compared to the DMSO control group, and only groups showing significance were identified. Statistically significant levels are indicated by asterisks, with *p < 0.05

Clustering of lipid rafts closely correlates with membrane lipid mobility. To investigate how CAG impacts lateral membrane fluidity, we measured the fluorescence recovery after photobleaching (FRAP) which represents a useful technique for studying the lipid mobility and diffusion of a given fluorophore-tagged molecule in lipid bilayers [29]. AGS cells were treated with each of different CAGs and analogues for 1 h, followed by GM1 labeling with the aforementioned Alexa Fluor 594-conjugated CT-b. Then a region of interest (ROI) with a 3 μm diameter was bleached with full laser power at 595 nm. Cell images were collected at 10 s thereafter and then taken at 20 s intervals for 290 s in total. Figure 2A shows a representative FRAP image sequence of AGS cells that were treated with CAG18:0 and then subjected to GM1 labeling. In contrast to the negative control (addition of DMSO), the treatment of CAG18:0 showed little or no fluorescence recovery in the entire time course after the ROI was photobleached. The FRAP recovery curves presented in Supplemental Fig. S1 with the mobile fraction calculated in Fig. 2B. *M*_f_ (mobile fraction) is defined by the lateral mobility of membrane lipids in a quantitative manner [30], designating to show the fraction of fluorescence recovering in the bleached region. *M*_*f*_ is calculated according to the following equation [19].

$${M}_{f}={[(F}_{\mu }- {F}_{0})/ {(F}_{Pre}- {F}_{0})]$$

${F}_{\mu }$ and $\mathrm{F}_{0}$ are denoted as the normalized fluorescence intensities showing indicated time events at 290 s and 0 s, respectively. ${F}_{Pre}$ is shown as the intensity level before the bleaching. The treatment of CAG18:0 significantly resulted in the lowest degree of membrane fluidity in AGS cells (Fig. 2B and Supplemental Fig. S1). In comparison with the aforementioned study of lipid rafts clustering, there was an inverse tendency about how an acyl chain of CAG affects the membrane mobility.

### Effect of an acyl chain in CAG on the *H. pylori* adhesion to gastric epithelium

**Fig. 3:**
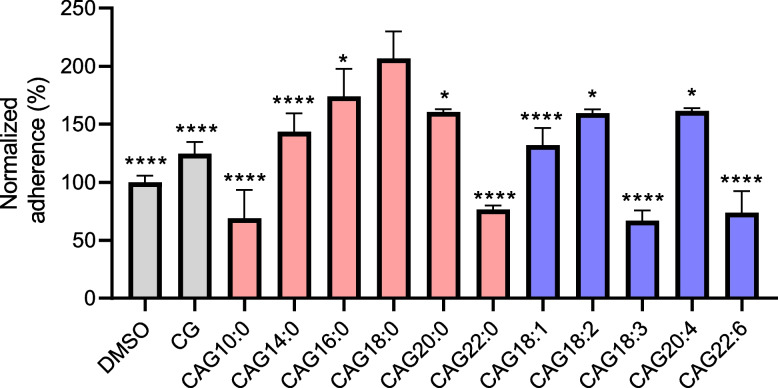
Treatments of AGS cells with CG and CAGs displayed a different degree of bacterial adhesion. AGS cells were first treated with CG and CAGs (as indicated) for 1 h, infected with *H. pylori* 26695 for another 1 h, and then subjected to flow cytometry analysis. Adherence was measured as the proportion of adhered AGS cells with *H. pylori*. One-way ANOVA with Dunnett's multiple comparisons test, comparing the control (DMSO and CG), and each treatment to the CAG18:0. Data represent mean ± SEM of biologically independent samples (*p < 0.05, **p < 0.01, ***p < 0.001, ****p < 0.0001)

We performed flow cytometric analysis to measure the extent of bacterial adhesion, to understand how acyl chains of various CAGs play a role. AGS cells were first treated with different CAGs or analogues for 1 h and then infected by *H. pylori* for another 1 h, followed by measuring the bacterial adhesion by flow cytometry [31] (Fig. 3). Similar to the results of lipid rafts clustering, the bacterial adhesion increased with a longer, saturated acyl chain of CAG when the chain length is below or equal to 18 carbons. But the trend was reversed once the chain length is above or equal to 18 carbons. In addition, regarding CAGs of unsaturated acyl chains, the treatment of AGS cells with CAG18:2 or CAG20:4 decreased the bacterial adhesion in comparison with that with CAG18:0. Among all the CAGs examined, CAG10:0, CAG18:3, CAG22:0, and CAG22:6 were the best to reduce the bacterial adhesion, as compared to CAG18:0 (Fig. 3), implying that these CAGs show the potential to diminish or prevent *H. pylori* adhesion.

### Treatment of* H. pylori *with PE(10:0)_2_, PE(18:3)_2_ or PE(22:6)_2_ reduced the bacterial adhesion to AGS cells

**Fig. 4:**
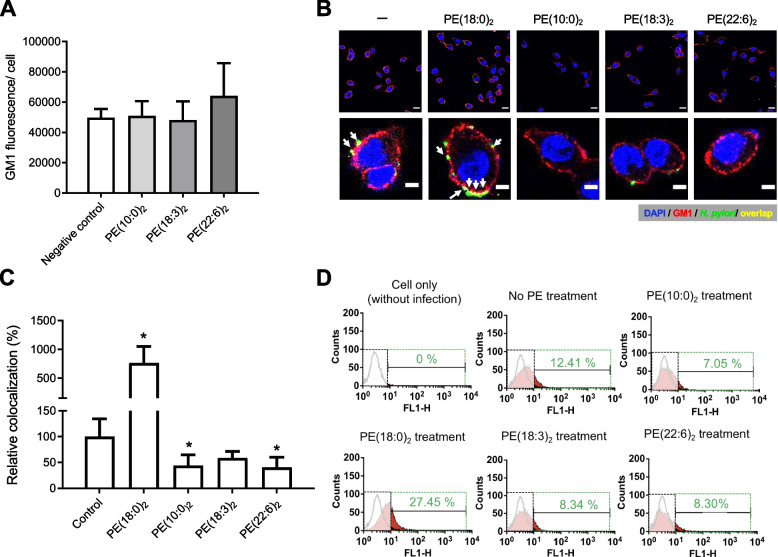
Treatment of with *H. pylori* cells with PE(10:0)_2_, PE(18:3)_2_ or PE(22:6)_2_ reduced their adhesion to AGS cells. **A** AGS cells were treated with PE(10:0)_2_, PE(18:3)_2_ or PE(22:6)_2_ for 1 h, followed by staining with Alexa Fluor 594-conjugated CT-b to measure the degree of lipid rafts clustering. Approximately 100 cells were measured in each experiment. **B ***H. pylori* was initially treated with PE containing specific acyl chains (as indicated) for 1 h and then cocultured with AGS cells for another 1 h. Lipid rafts clustering and nuclei of AGS cells were stained with CT-b (red fluorescence) and DAPI (blue), respectively. *H. pylori* was labeled by specific antibodies (green). Colocalization of *H. pylori* and lipid rafts clustering was indicated by white arrows (yellow). Scale bars: 10 (upper) and 5 (lower) μm. **C** Relative level of colocalization was quantified with ImageJ, calculated by co-localized signals per cell, and normalized according to that of the control (no PE-treated). Data represent the mean percentage of colocalization ± SEM (n ≥ 50 cells) and all statistically significant differences are indicated with asterisks; *p < 0.05 vs. the control group. **D ***H. pylori* was first treated with PE (as indicated) for 1 h and then cocultured with AGS cells for another 1 h. A degree of bacterial adhesion was then measured by flow cytometric analysis in accordance with the aforementioned procedure in Fig. 3

*H. pylori* has CGAT to catalyze the acyl transfer from PE to CG, leading to the formation of CAG [17]. We thus considered the possibility of converting specific PEs to corresponding CAGs in vivo, as an approach to prevent bacterial adhesion. However, we did not consider PE(22:0)_2_ because its abundance is extremely low in both human and *H. pylori* [32]. At first, we investigated whether the addition of PE resulted in direct alteration of any membrane features (e.g., lipid rafts clustering). AGS cells were treated with each of PE(10:0)_2_, PE(18:3)_2_ and PE(22:6)_2_ for 1 h, followed by measurement of lipid rafts clustering (shown by staining with Alexa Fluor 594-conjugated CT-b; see Fig. 4A). We observed either no obvious change (for PE(10:0)_2_ and PE(18:3)_2_), or slight increase (PE(22:6)_2_), as compared to the negative control (no PE added).

Furthermore, the cells of *H. pylori* were treated with PE(10:0)_2_, PE(18:0)_2_, PE(18:3)_2_ or PE(22:6)_2_ for 1 h and cocultured with AGS cells for 1 h. The level of bacterial infection was evaluated in accordance with the overlapped signals of lipid raft clustering (red) and *H. pylori* (green), as shown in Fig. 4B, and C. The result indicated that the prior treatment with PE(10:0)_2_, PE(18:3)_2_ or PE(22:6)_2_ significantly decreased lipid rafts clustering and the bacterial adhesion, in contrast to that with PE(18:0)_2_ (the positive control). Additionally, flow cytometric analysis was used to quantify the bacterial adhesion (Fig. 4D), in which the same conclusion was reached.

###  Treatment of *H. pylori *with PE(10:0)_2_ altered the lipid compositions of bacterial membranes

**Fig. 5:**
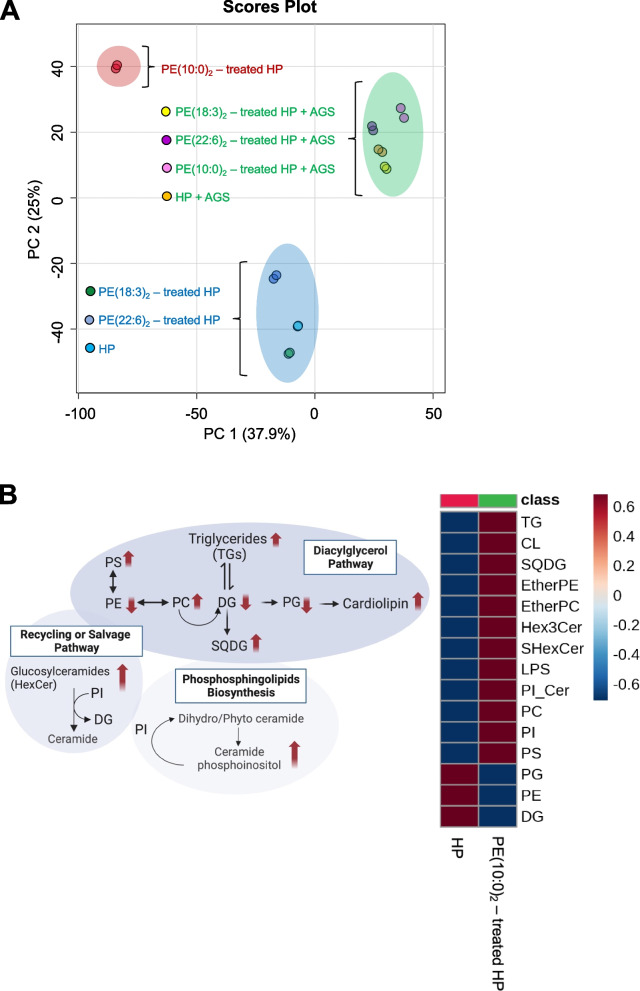
Lipidomics analysis of membrane lipids was performed in *H. pylori* treated with PE for 16 h. **A** Principal component analysis (PCA) was performed on the PE-treated *H. pylori* cultures and the bacterial cocultures with AGS cells. The resulting PCA score plot obtained from the data detected in positive mode MS analysis. Apparently there are two obvious clusters when comparing between the first principal component and the second. **B** The heat map displayed highly changed lipids in the *H. pylori* cells that were treated with or without PE(10:0)_2_. Changes in the lipid species were shown by colors ranging from positive correlation (red) to absence of correlation (white) and negative correlation (blue). A cutoff at FDR < 0.01 indicated 1% of all detected lipids resulted in false positives. Schematic diagram depicts up- or down-regulated lipids in PE(10:0)_2_-treated *H. pylori*. Abbreviations: PS (Phosphatidylserine), PE (Phosphatidylethanolamine), PI (Phosphatidylinositol), PC (Phosphatidylcholine), EtherPE (Ether-linked phosphatidylethanolamine), EtherPC (Ether-linked phosphatidylcholine), LPS (Lyso-PS), SQDG (Sulfoquinovosyl diacylglycerol), SHexCer (Sulfatide), and PI_Cer (ceramide phosphoinositol)

For the purpose of global profiling in the host and bacterial membranes, we performed untargeted lipidomics analysis on the aforementioned cell culture. The cells of *H. pylori* were treated with PE(10:0)_2_, PE(18:3)_2_, or PE(22:6)_2_ for 16 h. Subsequently, in the co-culture study, the PE-treated *H. pylori* cells were further co-incubated with AGS cells for an additional 1 h at 37 ℃. After the harvest, the cell mixtures were extracted with organic solvent, followed by LC–MS analysis. Principle component analysis (PCA) was conducted to assess the influence of PE treatment on membrane lipid compositions, as shown in Fig. 5A. Each data point within the scores plot represents the MS data obtained from a single test sample in the positive ion mode. This mode allows for the detection of the most lipid species that shaped *H. pylori* cell membranes, such as PE, phosphatidylglycerol (PG), and cardiolipin (CL). These lipids were detected in their intact molecular ion forms. There are two obvious clusters when comparing between the first principal component and the second. The PE-treated *H. pylori* cells (except for the PE(10:0)_2_-treated) converged into a unique group, and so did the PE-treated co-cultures of *H. pylori* and AGS cells. Each of the two groups produced very different membrane lipid profiles (Fig. 5A). Moreover, the lipids produced by the PE(10:0)_2_-treated *H. pylori* cells were found to greatly deviate from the aforementioned two groups, implicating that substantial disparities in the membrane compositions may help to explain the observed alterations in the aforementioned membrane features. Further heat map analysis was shown to highlight the lipid species likely associated with the remodeled membrane lipids (Fig. 5B). In this study, we observed sequential changes in diacylglycerols (DG), sulfoquinovosyl diacylglycerol (SQDG)), and ceramides, suggesting the exogenous PE(10:0)_2_ remodeled the membrane lipid compositions to prevent bacterial adherence to host cells. Furthermore, this study demonstrated the presence of two ether-linked phospholipids (EtherPE, EtherPC), potentially originating from static phospholipid pools. Additionally, other identified lipids, such as ceramide phosphoinositol (PI-Cer), were also reported. The observed changes were substantial (refer to Fig. 5B; Supplemental Fig. S4), representing a direct response to the PE(10:0)_2_ treatment.

Furthermore, the PE(18:0)_2_ treatment was also studied and compared because the treatment was previously shown to enhance the bacterial adhesion (Supplemental Fig. S2A). To understand what lipids were significantly changed between the negative control (*H. pylori* culture without PE treatment) and the PE(10:0)_2_-treated *H. pylori* cells, False Discovery Rate of 0.01 was applied to selectively identify more than 800 lipid metabolites of significant alterations, covering 59 lipid subclasses. These lipid subclasses mainly differ in their chain length, chain saturation, and functional group modification that were further grouped into six main lipid categories, such as sphingolipids (28.8%), glycerophospholipid (27.1%), glycerolipids (22.0%), sterol lipids, fatty acyls and prenol lipids (Supplemental Fig. S2B). Particularly, a vast majority of changes were observed in sphingolipids (including sulfonolipids, ceramide phosphoinositol, sulfatides (sulfated hexose ceramide)) and glycerophospholipids (including phosphatidylinositol (PI), PC, and PS) that are primarily responsible for driving this differentiation, as shown in Supplemental Fig. S2B. Lipids showing an increase with a fold change (FC) > 2.0 are regarded as strongly up regulated. Particularly, we observed an interesting trend when examining the acyl compositions of several abundant glycerophospholipids, including PS, PI, PC, Lyso-PS, Lyso-PI, and Lyso-PG. The percentage of very long chain fatty acids (VLCFA; containing > 48 total carbons in acyl chains) increased double (from 15.7% to 31.2%, see Supplemental Fig. S2C), whereas those of long chain fatty acid (LCFA, containing 28–48 carbons) and medium chain fatty acid (MCFA, 16–28 carbons) had small or little change (Supplemental Fig. S2C). Additionally, monounsaturated fatty acids (MUFA) and polyunsaturated fatty acids (PUFA) both increased in the analysis. VLCFA, MUFA and PUFA are all considered to increase membrane fluidity [33–36], preventing *H. pylori* from adhering to the host cells.

In previous studies, *H. pylori* cells were subjected to the PE treatment for 16 h. To study how shorter incubation impacted the lipid compositions, *H. pylori* cells were treated with PE(10:0)_2_ for 1 h and then co-cultured with AGS cells for another 1 h. PCA displayed three groups, including AGS cells, co-cultured AGS-*H. pylori* cells, and *H. pylori* cells (Supplemental Fig. S3A). No matter these cells were treated with or without PE(10:0)_2,_ the resulting data relatively converged. The heatmap in Supplemental Fig. S3B showed the changed lipid subclasses 1 h after the PE treatment, in which there are mainly sphingolipids (ceramides and derivatives) and glycerolipids including diacylglycerols (DG). Our analysis indicated the incorporation of the 10:0-acyl chain into various ceramides, diacylglycerides and their derived species, resulting from the uptake of PE(10:0)_2_ and the subsequent metabolic conversions in *H. pylori*. In this study, we elucidate the impact of PE(10:0)_2_ on lipid metabolism, particularly in the regulation of sphingolipid and diacylglycerol biosynthesis. The possibly involved pathways are illustrated in Supplementary Figure S3C.

###  The PE(10:0)_2_-containing diet significantly reduced* H. pylori *adhesion in a mouse model

We additionally investigated whether CGAT accepts PE(10:0)_2_, PE(18:3)_2_ or/and PE(22:6)_2_ to generate the corresponding CAGs. The cells of *H. pylori* 26695 were treated with PE(10:0)_2_, PE(18:3)_2_ or PE(22:6)_2_ at the final conc of 0.1 mM and cultured for 16 h. There was no obvious difference between these treated cells and the control (no PE added) when examining the bacterial morphology, growth and viability (data not shown). After harvest and the subsequent lysis, the amounts of various CAGs were quantified by LC–MS analysis (Supplemental Fig. S5). The result indicated that the formation of CAG reached to a plateau 4 h after the bacteria were treated with PE. Among the three PEs examined, PE(10:0)_2_ appeared to be the most compatible substrate for CGAT; CAG10:0 accounted for 58% of the total CAGs. On the other hand, PE(18:3)_2_ and PE(22:6)_2_ were less preferable for CGAT since the corresponding CAG18:3 (16%) and CAG22:6 (0.12%), respectively, were not major products.

**Fig. 6:**
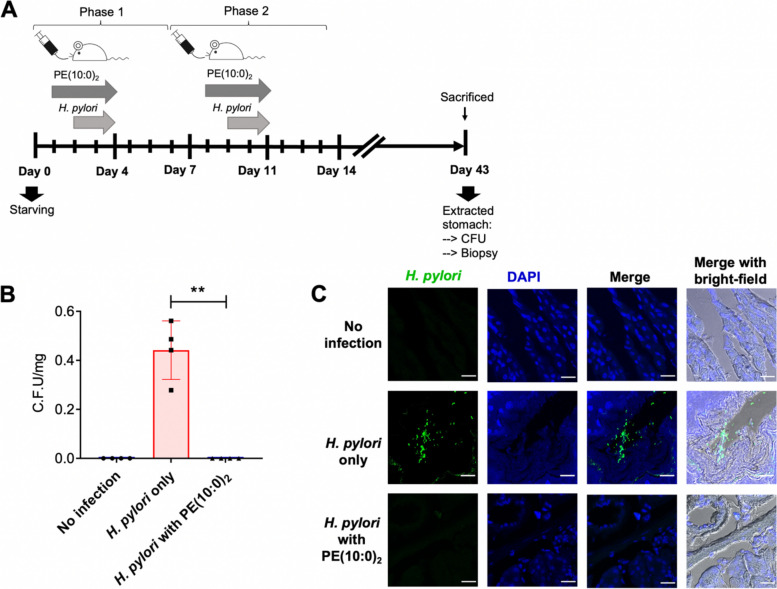
Feeding mice with the PE(10:0)_2_-containing diet abolished the bacterial adhesion. **A** A flowchart to explain the experimental procedure. B6 mice were starved for a day before they were treated with PE(10:0)_2_ and *H. pylori* infection for two phases. At day 1 of the first and second phases, mice were treated with 1000 mg/kg per mouse of PE(10:0)_2_ via oral gavage. At days 2, 3 and 4 of the two phases, these mice were co-treated via oral gavage with *H. pylori* (1 × 10^10^ cells) and 1000 mg/kg per mouse of PE(10:0)_2_. Mice were sacrificed at day 43 after the treatment procedure and the dissected gastric tissues were subjected to further analysis. **B** Degree of bacterial colonization was measured by colony-forming unit, i.e., identified bacterial colonies per mini-gram stomach tissue. Data are shown as mean ± SD (standard error) and all statistically significant differences are indicated with asterisks; ***p < 0.001, **p < 0.01, *p < 0.05 vs. the control group (*n* = 4). **C** The cells of *H. pylori* were detected by immunofluorescent staining in cryo-sections. After 4% formaldehyde fixation, the nuclei of murine gastric epithelial cells were shown in blue (by using Hoechst 33342 dye). The bacteria were labeled in green (by using *H. pylori*-specific monoclonal antibody and 488-conjugated secondary antibody). All images were acquired by confocal laser microscopy. The results shown are representative of four independent experiments. Scale bar: 20 μm

The idea of reducing or preventing bacterial infection was then examined in a mouse model. B6 mice were initially fed with 1000 mg/kg per mouse of PE(10:0)_2_ at day 1 (Fig. 6A) and subsequently treated with *H. pylori* (1 × 10^10^ cells) and 1000 mg/kg per mouse of PE(10:0)_2_ in 200 µl of culture medium at days 2, 3 and 4, followed by no such treatment for another three days. The same procedure was repeated once again in the following week. These mice were then sacrificed four weeks later [22, 23], the dissected gastric tissues were subjected to the colony-forming unit (CFU) assay for examining the degree of bacterial colonization [37, 38], i.e., to calculate the number of bacterial colonies per milligram of stomach tissue. *H. pylori* was detected in cryo-sections by using a specific monoclonal antibody. The confocal imaging analysis (Fig. 6C) indicated that bacterial adhesion was rarely found in the PE(10:0)_2_-treated group as compared to the control (buffer only, no PE-treated). The result of quantitation is shown in Fig. 6B. We previously demonstrated that multidrug-resistant strains of *H. pylori* contained a high level of CAG [17], which was realized likely due to the reduced membrane permeability. To examine whether the treatment of PE(10:0)_2_ is also effective against the infection by multidrug-resistant *H. pylori*, one multidrug-resistant strain of *H. pylori,* MDR4955 (collected from National Taiwan University Hospital), was first treated with PE(10:0)_2_ for 1 h and cocultured with AGS cells (MOI = 50) for another 1 h. The infected AGS cells were then fixed with 2% formaldehyde and subjected to flow cytometry analysis. To our delight, the PE(10:0)_2_-treated group displayed more than one half reduced adhesion, in comparison with the positive control (no PE-treated), as shown in Supplemental Fig. S6.

## Discussion

Bacterial outer membrane (OM) is a remarkable structure that serves as a protective barrier, shielding bacteria from a multitude of environmental stresses, including changes in temperature, pH, osmotic pressure, and exposure to antimicrobial agents. In response to these challenges, bacteria have evolved mechanisms to either remodel their membranes or alter the functions of OM proteins. We previously demonstrated that the CAG assembled in the host cells (i.e., CAG incorporating a host acyl chain) was able to enhance lipid rafts clustering and subsequently promote the bacterial adhesion [17]. In this work, efforts were further made to investigate how the acyl chain of CAGs influences membrane features. Being a rigid molecule, cholesterol can increase membrane packing. CAG and other forms of cholesteryl glucosides (including CG, CPG, and lyso-CPG) can be considered as the modified versions of cholesterol with the additional attachment of glucose, acyl, and/or phosphatidyl groups. The generation of cholesterol-derived lipids in *H. pylori* likely represents an evolved delicate way to fine-tune various features of membranes. CGAT is the bacterial enzyme to catalyze the acyl transfer from PE to CG, forming CAG. Since CGAT displays broad substrate specificity to accept various phospholipids (e.g., PE, PC, and PS) consisting of different acyl chains [17]. The product CAG’s acyl chain can thus have various chain length and a different degree of unsaturation.

We successfully presented the results indicating that membrane features can be controlled by the addition of a specific CAG or corresponding PE, representing an effective approach to either enhance or inhibit bacterial adhesion (Supplemental Fig. S7**)**. Manipulating the biosynthesis of CAG has shown a therapeutic potential to diminish the adhesion of *H. pylori,* taking advantage of CGAT’s broad specificity. Intriguingly, PE(10:0)_2_ was highly acceptable for CGAT, in contrast to PE(18:3)_2_ and PE(22:6)_2_, resulting in the main formation of CAG10:0. It explained why we only selected PE(10:0)_2_ for the subsequent mice study. Feeding mice with PE(10:0)_2_ was able to eradicate the bacterial adhesion in the mouse stomach. Meanwhile, we compared PE(10:0)_2_ with amiodarone that was previously shown to be a potent inhibitor against CGAT. *H. pylori* MDR4955 was treated with PE(10:0)_2_ or amiodarone for 1 h and then cocultured with AGS cells (MOI = 50) for another 1 h, followed by flow cytometry analysis to measure the degree of adhesion. As shown in Supplemental Fig. S8, the result indicated that PE(10:0)_2_ is slightly better than amiodarone to inhibit the bacterial adhesion. We additionally studied and compared how the treatment of PE(10:0)_2_ or amiodarone influenced the release of OMVs and vacuolating toxin (VacA) since both are critical for the pathogenesis and virulence of *H. pylori.* The treatment of PE(10:0)_2_ or amiodarone was found to influence the levels of secreted OMVs (Supplemental Fig. S9) and VacA (see Supplemental Fig. S10) in a different manner.

*H. pylori* leverages another strategy to modify its membrane, which was found critical for bacterial survival in the gastric mucosa. Cullen and coworkers prepared *H. pylori* mutants lacking lpxE (1-phosphatase to remove 1-phosphate of the lipid A in the bacterial OM), lpxF (4’-phsphatase), or lpxE/F (to remove both 1- and 4’-phosphates) that displayed a 16-, 360- and 1020-fold increase in sensitivity to the cationic antimicrobial peptide polymyxin B, respectively [39]. The action of LpxE and LpxF was able to decrease recognition of *H. pylori* LPS by the innate immune receptor, Toll-like Receptor 4. Their result indicated that dephosphorylation of the lipid A domain of *H. pylori* LPS by LpxE and LpxF determines its ability to colonize a mammalian host [39].

In this work, the *H. pylori* lipidome was examined by LC–MS analysis, following the PE(10:0)_2_ treatment of *H. pylori* culture. The result demonstrated that there were extraordinary changes in the lipid compositions, in addition to the formation of CAG10:0. Especially obvious alternations were found in glycerophospholipids and sphingolipids, which can be realized because their metabolic pathways are connected [40–42]. PCs, PIs, ether-linked phospholipids, and glycosphingolipids, usually existing as minor lipids in bacteria, were substantial lipid components in our analysis (Fig. 5). Human commensals, such as *Bacteroides* and *Porphyromonas,* were reported to produce ceramide phosphoinositols as one main component in their membranes [43], playing a role in regulating the gut homeostasis [44]. In this work, we observed an increase in the levels of several lipid subclasses containing 10:0-acyl chain, particularly in ceramides, and DGs, within the first incubation hour of *H. pylori* with PE(10:0)_2_. Additionally, PE(10:0)_2_ was shown to significantly decrease at the same time (see the top right subfigure in Supplemental Fig. S3C), suggesting its direct efficient uptake by *H. pylori.* Notably, the incorporation of 10:0-acyl chain led to the formation of not only CAG10:0, but also other dihydro- and phyto-sphingosine derivatives and ceramides. Interestingly, when the treatment duration was extended (i.e., 16 h), the aforesaid effect of 10:0-acyl chain was no longer observed. Instead, there were evident increases in VLCFA-, MUFA- and PUFA-components in several abundant subgroups of glycerophospholipids (Supplemental Figures S2B, C). Although we are not clear about potential impact of the extended length and degree of unsaturation, these unusual acyl chains may represent a cellular response to the exogeneous PE(10:0)_2_ treatment. It is acknowledged that the presence of polyunsaturated fatty acids (PUFA) serves as a determinant influencing both bacterial growth [45] and their attachment to the host cell's plasma membrane [46].

The occurrence of acyl-chain remodeling was also reported in the anti-microbial resistance of other Gram-negative bacteria. For example, *Salmonellae* PhoPQ system regulates the incorporation of aminoarabinose, ethanolamine, and phosphate to lipid A [47, 48]. Recently, Tao and coworkers reported their lipidomics studies on colistin-exposed multidrug-resistant *Acinetobacter baumannii*, where the significant changes included PE, PG, lyso-PE, hemibismonoacylglycerophosphate, CL, monolyso-CL, diacylglycerol, and triacylglycerol [49]. Interestingly, the majority of these lipid alterations coincide with what were identified in our analysis. Moreover, PagP from *Salmonella Typhimurium* serving as a phospholipase/acyltransferase [50, 51] under the regulation of PhoPQ, has been recognized for its dual functionality: transferring the palmitoyl chain to both phosphatidylglycerol (contributing to the outer membrane lipid homeostasis) [48] and lipid A (conveying the resistance against antimicrobial peptides) [52]. In addition, elevated levels of cardiolipins (CLs) [53] and palmitoylated acylphosphatidylglycerols [54] were found within the outer membrane of *Salmonellae Typhimurium*, serving as the mechanism through which the PhoPQ system modulates the acidic phospholipids [48] to impart resistance against cationic antimicrobial peptides [52, 55].

The idea of using a PE-containing diet is simple, straightforward, and practical. Since the approach is not bactericidal, the bacteria do not have to develop resistant mechanisms. Moreover, most PEs, including PE(10:0)_2_ and other PEs in this study, are commercially available at low cost and can be purified at a large scale by known procedures. For example, PE in raw milk accounts for 22.6% [56] to 42% [57, 58] of the total phospholipids. Capric acid (10:0) was found to be one of the major fatty acids in goat milk [59, 60]. Moreover, other lipids can be probably considered as alternative sources. Phospholipid biosynthesis is a complex process because it contains many branch points. Phosphatidic acid (PA) plays a central role in biosynthesis since PA [61] is the precursor of all phospholipids synthesized (including PE) via the formation of cytidine diphosphate–diacylglycerol (CDP–DAG) [62, 63]; i.e., PA is converted to CDP–DAG, which is then utilized for the synthesis of PE, PC, and PS [64–66]. There are two major pathways to produce PE: the PS decarboxylation pathway (in which PS is directly decarboxylated to produce PE) and the CDP-ethanolamine pathway[62]. In the latter pathway, PE is made by the coupling of CDP-ethanolamine with diacylglycerol (which can be derived from PA). As a consequence, PS(10:0)_2_ and PA(10:0)_2_ are likely suitable substitutes of PE(10:0)_2_.

We are still puzzled about how the treatment of with PE(18:3)_2_ or PE(22:6)_2_ led to inhibited bacterial adhesion (Fig. 4B, D). Our MS analysis indicated that there was limited formation of corresponding CAG18:3 or CAG22:6. Instead, CAG14:0 existed predominantly in the two treated groups. Finding a possible explanation is likely challenging since the PE treatment impacted the membranes of AGS cells and *H. pylori* at the same time. Eukaryotic and prokaryotic cells are known to leverage different mechanisms to regulate membrane lipid homeostasis [48, 67]. Apparently, there are other mechanisms orchestrating the inhibition.

## Conclusion

In conclusion, we discovered that rewired CAG biosynthesis led to the remodeling of membrane lipid compositions. The usage of PE (as CAG’s precursor) successfully inhibited the adhesion of *H. pylori* and eradicated the bacteria in the mouse stomach. Since the blockade was also effective to reduce the adhesion of multidrug-resistant *H. pylori*, the idea of PE treatment, representing an easy and effective way for tackling the challenge of drug resistance, indeed displays great potential for further therapeutic development.

### Supplementary Information

**Additional file 1: Supplemental Figure S1.** Quantitative FRAP analysis of AGS cells that were treated with CAGs of various acyl chains. AGSs cells were first treated with CG or each of CAGs, and then subjected to FRAP experiments as described in Fig. 2. Each of normalized red fluorescence recovery curves showed the relative mean intensity measured from five cells. (A) Curves correspond to the treatments of AGS cells with CAGs containing saturated acyl chains. (B) Curves correspond to the treatments of AGS cells with CAG18:0, CAG18:1, CAG18:2 and CAG18:3.

**Additional file 2: Supplemental Figure S2.** Significant changes in the lipid subclasses in response to the 16-h PE(10:0)_2_ treatment.(A) Principal component analysis (PCA) was performed for the PE-treated *H. pylori* cultures and the cocultures of *H. pylori*–AGS cells. The cells of *H. pylori* were treated with PEs for 16 h. For the purpose of coculture, the previously mentioned *H. pylori *cells were further cocultured with AGS cells for another 1 h. The resulting PCA score plot were obtained from the data detected in the positive mode of MS analysis. Apparently there are two obvious clusters when comparing between the first principal component and the second. (B) Integrated circular dendrogram to show significant changes in lipid subclasses resulting from the 16-h PE(10:0)_2_ treatment. All the lipids with FDR<0.01 were classified into various lipid subclasses (e.g., DG, TD, PE, PC, PI, Cer_NDS (ceraminde non-hydroxyfatty acyl dihydrosphingosine)) that were subsequently grouped together into six lipid classes (e.g., glycerolipids (GL), glycerophospholipids (GP) and fatty acyls (FA)), shown in different colors. The size of nodes represents the counts of each individual lipid subclass to indicate its diversity (e.g., different chain length, saturation). (C) Acyl compositions of the six most abundant glycerophospholipids. The cells of *H. pylori* were treated with PE(10:0)_2_ for 16 h, followed by lipidomic analysis. Individual lipids from six abundant glycerophospholipid subclasses, including PS, PI, PC, LPS, LPI and LPG, were grouped and then divided into two categories (i.e., decrease lipids (FC<1) and increase lipids (FC>1)) for characterization. For lipid chain length (shown in blue gradient), the lipids were characterized by short chain fatty acids (SCFAs, 4-16 carbons in total), medium chain fatty acids (MCFAs, 16-28 carbons), long chain fatty acids (LCFAs, 28-48 carbons), and very long chain fatty acids (VLCFAs, >48 carbons). For lipid saturation (shown in green gradient), the lipids were characterized by saturated fatty acids (SFAs, no double bond), monounsaturated fatty acids (MUFAs, one double bond), di-unsaturated fatty acids (DUFAs, two double bonds), and polyunsaturated fatty acids (PUFAs).

**Additional file 3: Supplemental Figure S3.** Significant changes were found in the lipid subclasses in response to the 1-h PE(10:0)_2_ treatment. (A) Principal component analysis (PCA) was performed for the PE(10:0)_2_-treated *H. pylori* culture and the coculture of *H. pylori*–AGS cells. The cells of *H. pylori* were treated with PE(10:0)_2_ for 1 h. For the purpose of coculture, the previously mentioned *H. pylori *cells were further cocultured with AGS cells for another 1 h. The resulting PCA score plot were obtained from the data detected in the positive mode of MS analysis. Apparently there are two obvious clusters when comparing between the first principal component and the second. (B) In the heatmaps, the x axes show the *H. pylori cell *control groups vs. PE(10:0)_2_-treated *H. pylori* groups. The heat map displayed highly changed lipids in the *H. pylori* cells that were treated with or without PE(10:0)_2_ for 1 h at 37 ^o^C, and then cocultured with AGS cells (MOI = 50). Changes in the lipid species were shown by colors ranging from positive correlation (red) to the absence of correlation (white) and negative correlation (blue). A cutoff at FDR < 0.01 indicated 1% of all detected lipids resulted in false positives. (C) The schematic diagram showed the predicted remodeling lipids biosynthesis pathways in response to 1-h PE(10:0)_2_ treatment on *H. pylori*, depicting the pathways of ceramide metabolism significant upregulation of lipids, including Cer 12:0;O2/10:0;O (FC = 170.2), Cer 12:0;O2/10:0 (FC = 1.7), and Cer 12:1;O2/10:0;O (FC = 239.7), in response to the PE(10:0)_2_ treatment. Additionally, other lipid groups, such as diacylglycerol (DG 10:0), were found to increase. A decrease was observed in lipid groups incorporating with 10:0 or short chain are mostly derived from sphingosine Cer.12:1; O3/10:0/2OH, lysophosphatidylcholines LPC 10:0, sphingomyelin SM 12:1; O2/10:0, and sulfoquinovosyl-diacylglycerol SQDG 10:0/8:0 (****p < 0.0001).

**Additional file 4: Supplemental Figure S4.** Global lipidome changes: a heatmap of the top 25 significantly altered lipid classes in response to the PE(10:0)_2_ treatment for 1 h.For Cells group and Cells_HP group: Lipid classes belonging to Group 1 (G1) and Group 2 (G2) were identifiable but absent in both the HP and HP_PE bacteria-containing groups, indicating that these lipids undergo changes in response to PE(10:0)_2_ treatment. Lipid classes categorized under Group 3 (G3) exhibited significant alterations in the PE(10:0)_2_ treatment groups for cell-only groups (cells_PE or HP_PE). However, in the co-culture group (Cells_HP), these lipids decreased, indicating that they were not transferred between each cell type. Lipid classes marked with an asterisk (*) likely originated from bacteria-containing groups, but these lipids were not among data from our two batch experiment (top 25 highly regulated lipids in 1-hr-PE(10:0)_2_-treament batch experiment and top 15 highly regulated lipids in 16-hr-PE(10:0)_2_-treament batch experiment) after applying a false discovery rate (FDR) cutoff of 0.01. Changes are color coded; numbers indicate fold-changes. The x axes show experimental groups: Cells (AGS cells only); Cells_HP (AGS cells with H. pylori infection); Cells_HP_PE (AGS cells with PE(10:0)_2_-treated *H. pylori*); Cells_PE (AGS cells with PE(10:0)_2_-treatment); HP only (*H. pylori* bacterial cells only); HP_PE (*H. pylori* with PE(10:0)_2_-treatment). The y axes show lipids classes with abbreviations: MG (Monoacylglycerol); CoQ (Coenzyme Q); NAGlySer (N-acyl glycyl serine); LPS (Lysophosphatidylserine); PI_Cer (Ceramide phosphatidylinositol); LDGTS (Lysodiacylglyceryl trimethylhomoserine); EtherLPE (Ether-linked lysophosphatidylethanolamine); Unknown (Unidentified lipid); PhytoSph (Phytosphingosine); DHSph (Sphinganine); NAGly (N-acyl glycine); BASulfate (Cholic acid sulfate); PS (Phosphatidylserine); PE (Phosphatidylethanolamine); PC (Phosphatidylcholine); Cer_HS (Ceramide hydroxy fatty acid-sphingosine); CASE (Campesterol ester); HexCer_NS (Hexosylceramide non-hydroxyfatty acid-sphingosine); CerP (Ceramide 1-phosphates); LPC (Lysophophatidylcholine); EtherPE (Ether-linked phosphatidylethanolamine); ST (Sterols); CAR (Carnitines); SSulfate (Sterol sulfate); PG (Phosphatidylglycerol).

**Additional file 5: Supplemental Figure S5. **Quantitative analysis of theconversion of PEs to corresponding CAGs in H. pylori.(A) A flowchart of measuring the conversion of PEs to corresponding CAGs in *H. pylori*. 17β-([3’-Azidopropoxy)-5-androsten-3β-ol (an azide-containing analogue of cholesterol) was added to *H. pylori* cells culture medium (at the final conc of 50 µM) and incorporated into the biosynthetic pathway to produce the analogues of CAGs and other cholesteryl glucoside derivatives. PE(10:0)_2_, PE(18:3)_2_ or PE(22:6)_2_ was also added to the bacterial culture for 0.5, 1, 2, 4, 8, and, 16 h. After harvest and Folch extraction, these compounds were further reacted with a fluorescent alkyne (4-*N*-methylamino-1,8-napthalimidopropyne) via Cu(I)-catalyzed 1,3-dipolar cycloaddition to obtain the fluorophore-conjugated products. The resulting products were analyzed by UPLC-MS. (B) Ratios of specific CAGs to total CAGs were determined by analyzing the area of product ion signals in the UPLC-MS spectra. The levels of the incorporated CAGs (CAG10:0, CAG18:3, or CAG22:6) relative to total CAGs were measured at 0.5, 1, 2, 4, 8, and 16 h after treatment with PE(10:0)_2_, PE(18:3)_2_ or PE(22:6)_2_. The data were obtained from three biological replicates. (C) Pi-charts display percentages of various CAGs that were analyzed after *H. pylori* was treated with precursor PE(10:0)_2_, PE(18:3)_2_ or PE(22:6)_2_ for 16 h.

**Additional file 6: Supplemental Figure S6.** Effect of PE(10:0)_2_ to inhibit the adhesion of the multidrug resistant H. pylori to AGS cells. The MDR strain of *H. pylori *MDR4955 was first treated with PE(10:0)_2_ for 1 h and then cocultured with AGS cells (MOI = 50) for another 1 h. The cells were detached from plates by using trypsin after washes with Dulbecco’s phosphate-buffered saline for three times, fixed with 2% formaldehyde, and then subjected to flow cytometry analysis. *H. pylori-s*pecific antibody (Abcam, ab20459, 1:1000) was used. Adherence was measured as the proportion of adhered AGS cells with *H. pylori*. The quantification of cell adhesion was normalized relative to the highest group, which was the "Cell with MDR infection," set as 100%. Representative data are shown as mean ± SD (standard error) (*n* = 6). Statistical analysis was performed using Student's *t* test with *p*-values less than 0.05 shown as one asterisk. Cell + MDR, cell infected by MDR4955; Cell + MDR-PE, cell infected by PE(10:0)_2_ - treated MDR4955.

**Additional file 7: Supplemental Figure S7. **A proposed model to explain how the acyl chain affects membrane dynamics. A proposed model explaining how CAGs containing different acyl chains (e.g., 18:0, 10:0, 18:3, 22:6) remodel the cell membrane to either enhance or reduce bacterial adhesion. Created with BioRender.com.

**Additional file 8: Supplemental Figure S8.** Comparison of PE(10:0)_2_ and amiodarone in the bacterial adhesion.One multi-drug resistant strain of *H. pylori* MDR4955 was treated with PE(10:0)_2_ (100 µM) or amiodarone (50 µM) for 1 h and then cocultured with AGS cells (MOI = 50) for another 1 h. The resulting cells were detached from plates by using trypsin, washed with Dulbecco’s phosphate-buffered saline for three times, fixed with 2% formaldehyde, and subsequently subjected to flow cytometry analysis. *H. pylori*-specific antibody (Abcam, ab20459, 1:1000) was used for detecting the bacterial adhesion. The degree of cell adhesion was normalized relative to the highest group (AGS cells infected with MDR4955), set as 100%. Adherence was shown as the proportion of adhered AGS cells with *H. pylori*. Representative data are shown as mean ± SD (standard deviation) (*n*=3 for the groups treated with PE(10:0)_2_ and *n*=2 for the groups treated with amiodarone). Statistical analysis was performed using unpaired t test with Welch’s correction. *p*-Value is less than 0.05 shown as one asterisk, and less than 0.01 shown as two asterisks. Abbreviations: Cell + MDR, AGS cells cocultured with MDR4955; Cell + MDR + Ami, AGS cells cocultured with MDR4955 that were pretreated with amiodarone; Cell + MDR + PE10, AGS cells cocultured with MDR4955 that were pretreated with PE(10:0)_2_.

**Additional file 9: Supplemental Figure S9.** Effect of PE(10:0)_2_ or amiodarone on the OMVs secreted. The cells of *H. pylori *26695 were treated with either PE(10:0)_2_ (100 µM; results shown by red color) or amiodarone (50 µM; results by green color) for 3 days. The level of secreted OMVs from *H. pylori* were monitored at 24 h, 48 h and 72 h. Secreted OMVs were isolated according to previous procedure, followed by labelling with a fluorescent lipophilic dye, FM4-64. The OMV level was quantified by detecting the corresponding excitation/emission at 515/640 nm and then normalized on the basis of cell number. Representative data are shown as mean ± SD (standard deviation) (*n* ≥ 2). Abbreviation: HP only, *H. pylori* with no treatment; HP + PE, *H. pylori* treated with PE(10:0)_2_; HP + Ami, *H. pylori* treated with amiodarone.

**Additional file 10: Supplemental Figure S10.** The cells of *H. pylori* 26695 were treated with either PE(10:0)_2_ (100 µM) or amiodarone (50 µM), followed by isolating the secreted OMVs to detect the VacA level at 24 h, 48 h and 72 h.(A) Western blot analysis of VacA protein. Abbreviations: H, *H. pylori* with no treatment; P, *H. pylori* treated with PE(10:0)_2_; A, *H. pylori* treated with amiodarone. ‘No-stain’ labelling was used as the loading control for the purpose of quantification. The quantification of VacA levels was normalized relative to the first 24 h of *H. pylori* control group, set as 100%. (B) Relative VacA level was measured by quantifying the density of each band and normalized according to the density of the corresponding total proteins (namely ‘No-stain’ labelling). Summarized quantification data from independent western blots (*n*=4) are shown as mean ± S.E.M. (standard error of the mean). **** *P* < 0.0001 vs. control, *** *P* < 0.001 vs. control, ** *P* < 0.01 vs. control (*H. pylori* only in each group at 24, 48 or 72 h). Statistical analyses were performed using two-way analysis of variance (ANOVA).

**Additional file 11. **^1^H and ^13^C NMR spectra of CAGs (see Appendixes)

**Additional file 12.**

## Data Availability

The relevant data and its supplemental data can be found in the article or obtained from the corresponding author upon request.

## Abbreviations

AGS cells: Human gastric adenocarcinoma cell-line
MDR: Multi-drug resistant
PC: Phosphatidylcholine
PE: Phosphatidylethanolamine
PI: Phosphatidylinositol
PS: Phosphatidylserine
SCFAs: Short chain fatty acids
MCFAs: Medium chain fatty acids
LCFAs: Long chain fatty acids
VLCFAs: Very long chain fatty acids
SFAs: Saturated fatty acids, no double bond
MUFAs: Monounsaturated fatty acids, one double bond
DUFAs: Di-unsaturated fatty acids, two double bonds
PUFAs: Polyunsaturated fatty acids
FC: Fold change
