# Supplementary figures and images for: Membrane lipid remodeling eradicates Helicobacter pylori by manipulating the cholesteryl 6'-acylglucoside biosynthesis
Source: J Biomed Sci. 2024 Apr 29;31:44. doi: 10.1186/s12929-024-01031-8 (PMC11057186; doi:10.1186/s12929-024-01031-8)

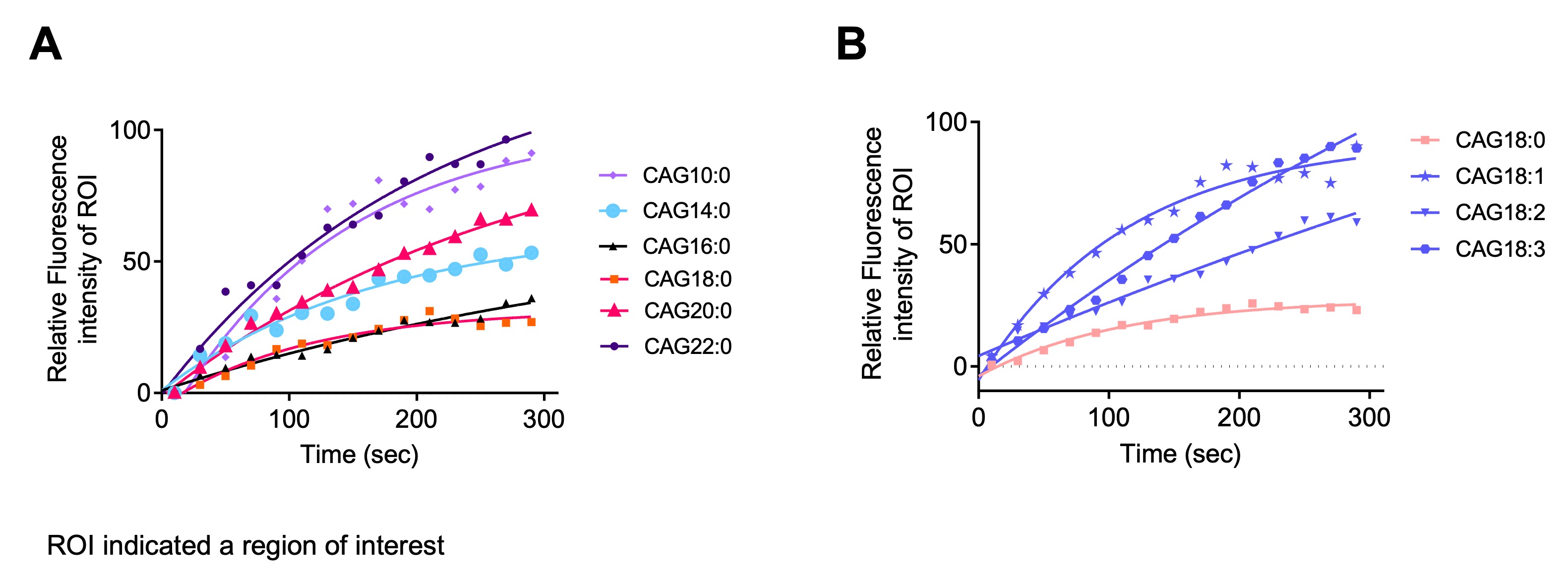

Supplement: Supplementary file 1 — Additional file 1: Supplemental Figure S1. Quantitative FRAP analysis of AGS cells that were treated with CAGs of various acyl chains. AGSs cells were first treated with CG or each of CAGs, and then subjected to FRAP experiments as described in Fig. 2. Each of normalized red fluorescence recovery curves showed the relative mean intensity measured from five cells. (A) Curves correspond to the treatments of AGS cells with CAGs containing saturated acyl chains. (B) Curves correspond to the treatments of AGS cells with CAG18:0, CAG18:1, CAG18:2 and CAG18:3. [file 12929_2024_1031_MOESM1_ESM.jpg]

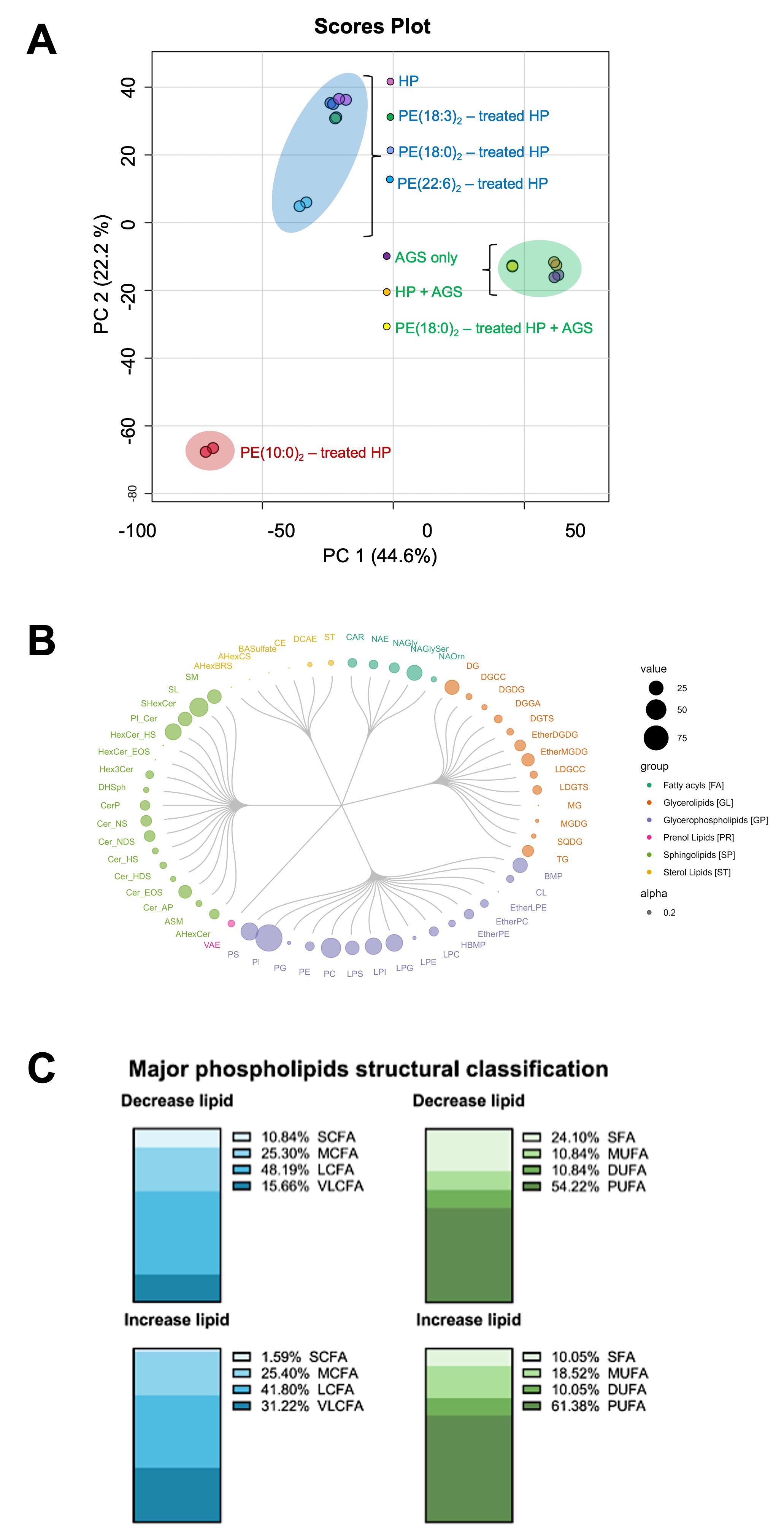

Supplement: Supplementary file 2 — Additional file 2: Supplemental Figure S2. Significant changes in the lipid subclasses in response to the 16-h PE(10:0)2 treatment.(A) Principal component analysis (PCA) was performed for the PE-treated H. pylori cultures and the cocultures of H. pylori–AGS cells. The cells of H. pylori were treated with PEs for 16 h. For the purpose of coculture, the previously mentioned H. pylori cells were further cocultured with AGS cells for another 1 h. The resulting PCA score plot were obtained from the data detected in the positive mode of MS analysis. Apparently there are two obvious clusters when comparing between the first principal component and the second. (B) Integrated circular dendrogram to show significant changes in lipid subclasses resulting from the 16-h PE(10:0)2 treatment. All the lipids with FDR<0.01 were classified into various lipid subclasses (e.g., DG, TD, PE, PC, PI, Cer_NDS (ceraminde non-hydroxyfatty acyl dihydrosphingosine)) that were subsequently grouped together into six lipid classes (e.g., glycerolipids (GL), glycerophospholipids (GP) and fatty acyls (FA)), shown in different colors. The size of nodes represents the counts of each individual lipid subclass to indicate its diversity (e.g., different chain length, saturation). (C) Acyl compositions of the six most abundant glycerophospholipids. The cells of H. pylori were treated with PE(10:0)2 for 16 h, followed by lipidomic analysis. Individual lipids from six abundant glycerophospholipid subclasses, including PS, PI, PC, LPS, LPI and LPG, were grouped and then divided into two categories (i.e., decrease lipids (FC<1) and increase lipids (FC>1)) for characterization. For lipid chain length (shown in blue gradient), the lipids were characterized by short chain fatty acids (SCFAs, 4-16 carbons in total), medium chain fatty acids (MCFAs, 16-28 carbons), long chain fatty acids (LCFAs, 28-48 carbons), and very long chain fatty acids (VLCFAs, >48 carbons). For lipid saturation (shown in green gradient), [file 12929_2024_1031_MOESM2_ESM.jpg]

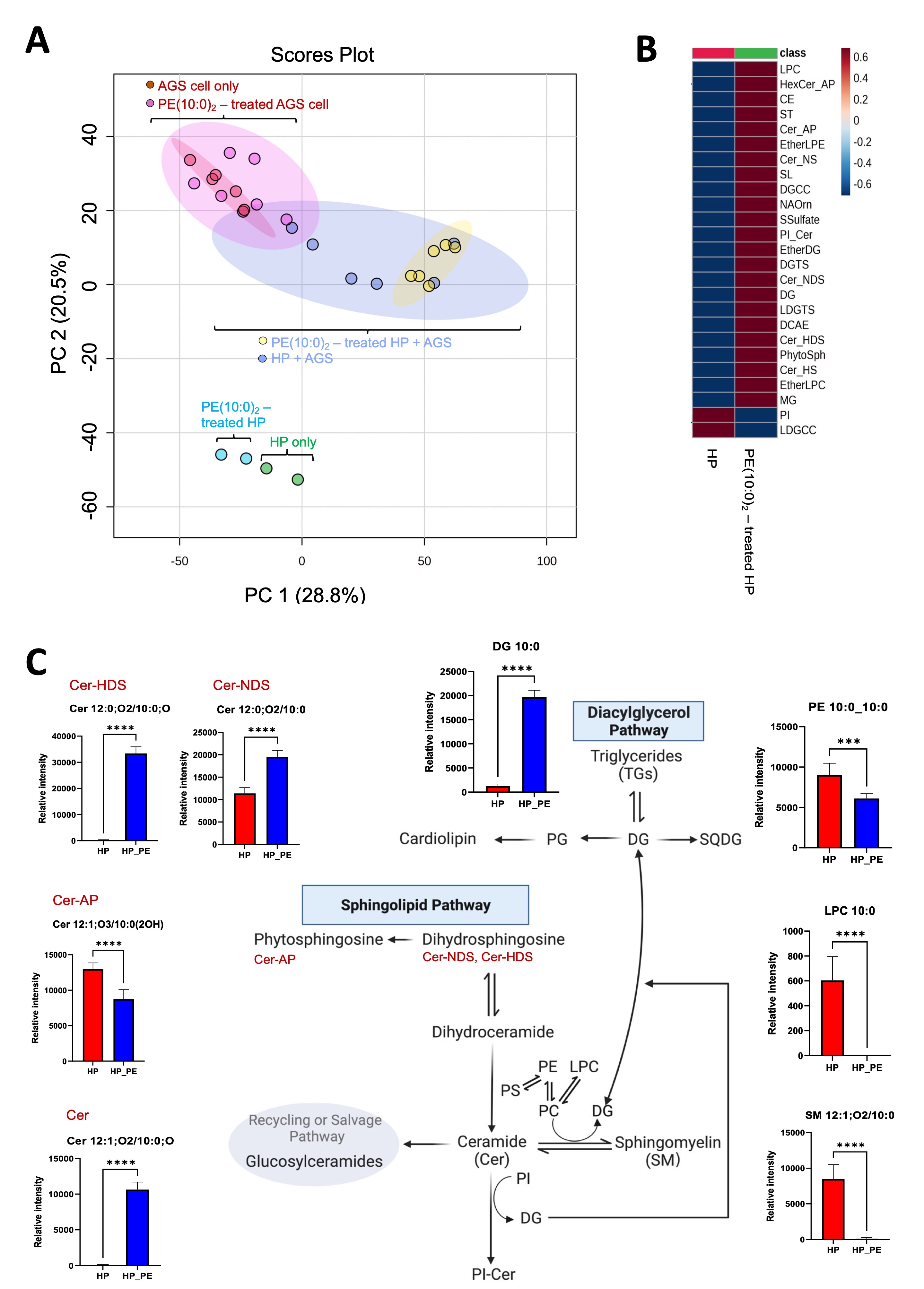

Supplement: Supplementary file 3 — Additional file 3: Supplemental Figure S3. Significant changes were found in the lipid subclasses in response to the 1-h PE(10:0)2 treatment. (A) Principal component analysis (PCA) was performed for the PE(10:0)2-treated H. pylori culture and the coculture of H. pylori–AGS cells. The cells of H. pylori were treated with PE(10:0)2 for 1 h. For the purpose of coculture, the previously mentioned H. pylori cells were further cocultured with AGS cells for another 1 h. The resulting PCA score plot were obtained from the data detected in the positive mode of MS analysis. Apparently there are two obvious clusters when comparing between the first principal component and the second. (B) In the heatmaps, the x axes show the H. pylori cell control groups vs. PE(10:0)2-treated H. pylori groups. The heat map displayed highly changed lipids in the H. pylori cells that were treated with or without PE(10:0)2 for 1 h at 37 oC, and then cocultured with AGS cells (MOI = 50). Changes in the lipid species were shown by colors ranging from positive correlation (red) to the absence of correlation (white) and negative correlation (blue). A cutoff at FDR < 0.01 indicated 1% of all detected lipids resulted in false positives. (C) The schematic diagram showed the predicted remodeling lipids biosynthesis pathways in response to 1-h PE(10:0)2 treatment on H. pylori, depicting the pathways of ceramide metabolism significant upregulation of lipids, including Cer 12:0;O2/10:0;O (FC = 170.2), Cer 12:0;O2/10:0 (FC = 1.7), and Cer 12:1;O2/10:0;O (FC = 239.7), in response to the PE(10:0)2 treatment. Additionally, other lipid groups, such as diacylglycerol (DG 10:0), were found to increase. A decrease was observed in lipid groups incorporating with 10:0 or short chain are mostly derived from sphingosine Cer.12:1; O3/10:0/2OH, lysophosphatidylcholines LPC 10:0, sphingomyelin SM 12:1; O2/10:0, and sulfoquinovosyl-diacylglycerol SQDG 10:0/8:0 (****p < 0.0001). [file 12929_2024_1031_MOESM3_ESM.jpg]

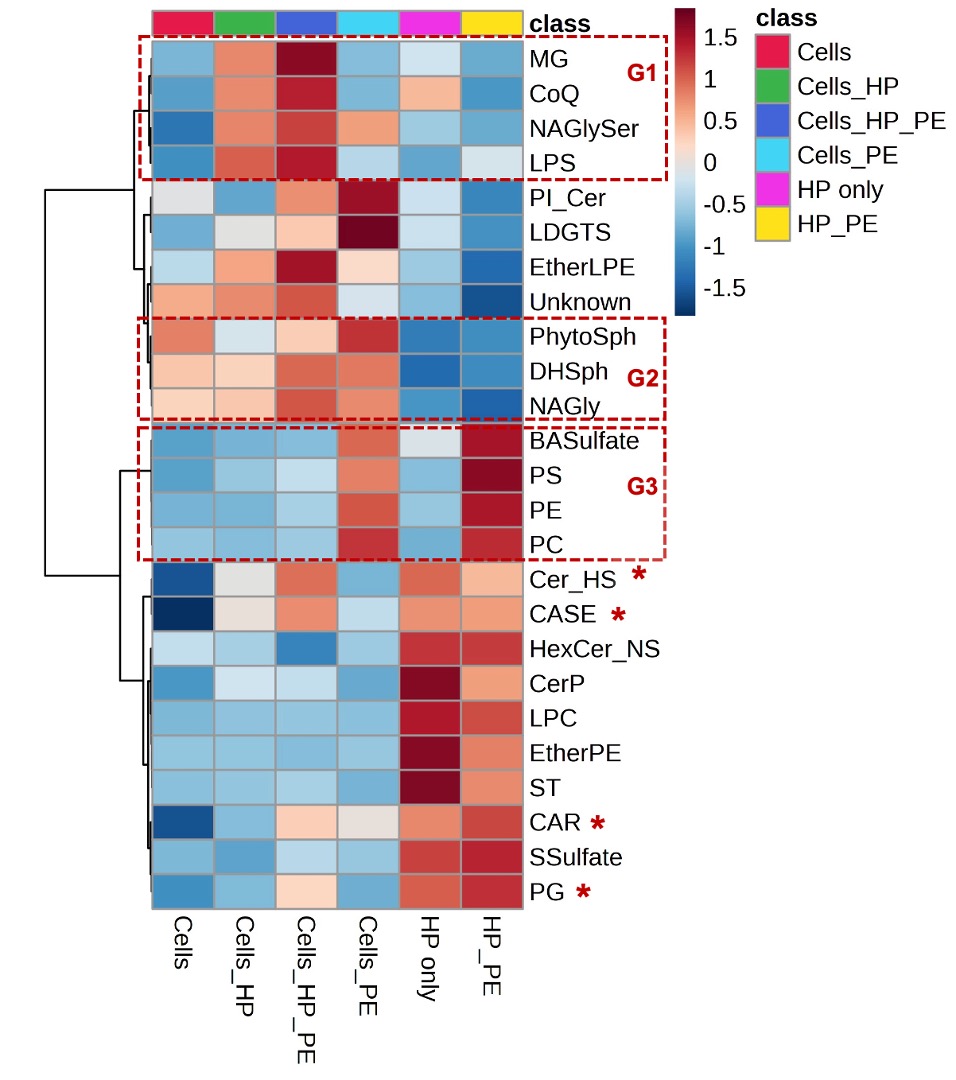

Supplement: Supplementary file 4 — Additional file 4: Supplemental Figure S4. Global lipidome changes: a heatmap of the top 25 significantly altered lipid classes in response to the PE(10:0)2 treatment for 1 h.For Cells group and Cells_HP group: Lipid classes belonging to Group 1 (G1) and Group 2 (G2) were identifiable but absent in both the HP and HP_PE bacteria-containing groups, indicating that these lipids undergo changes in response to PE(10:0)2 treatment. Lipid classes categorized under Group 3 (G3) exhibited significant alterations in the PE(10:0)2 treatment groups for cell-only groups (cells_PE or HP_PE). However, in the co-culture group (Cells_HP), these lipids decreased, indicating that they were not transferred between each cell type. Lipid classes marked with an asterisk (*) likely originated from bacteria-containing groups, but these lipids were not among data from our two batch experiment (top 25 highly regulated lipids in 1-hr-PE(10:0)2-treament batch experiment and top 15 highly regulated lipids in 16-hr-PE(10:0)2-treament batch experiment) after applying a false discovery rate (FDR) cutoff of 0.01. Changes are color coded; numbers indicate fold-changes. The x axes show experimental groups: Cells (AGS cells only); Cells_HP (AGS cells with H. pylori infection); Cells_HP_PE (AGS cells with PE(10:0)2-treated H. pylori); Cells_PE (AGS cells with PE(10:0)2-treatment); HP only (H. pylori bacterial cells only); HP_PE (H. pylori with PE(10:0)2-treatment). The y axes show lipids classes with abbreviations: MG (Monoacylglycerol); CoQ (Coenzyme Q); NAGlySer (N-acyl glycyl serine); LPS (Lysophosphatidylserine); PI_Cer (Ceramide phosphatidylinositol); LDGTS (Lysodiacylglyceryl trimethylhomoserine); EtherLPE (Ether-linked lysophosphatidylethanolamine); Unknown (Unidentified lipid); PhytoSph (Phytosphingosine); DHSph (Sphinganine); NAGly (N-acyl glycine); BASulfate (Cholic acid sulfate); PS (Phosphatidylserine); PE (Phosphatidylethanolamine); PC (Phosphatidylcholine); Cer_HS (Ceramide hydroxy fatty [file 12929_2024_1031_MOESM4_ESM.jpg]

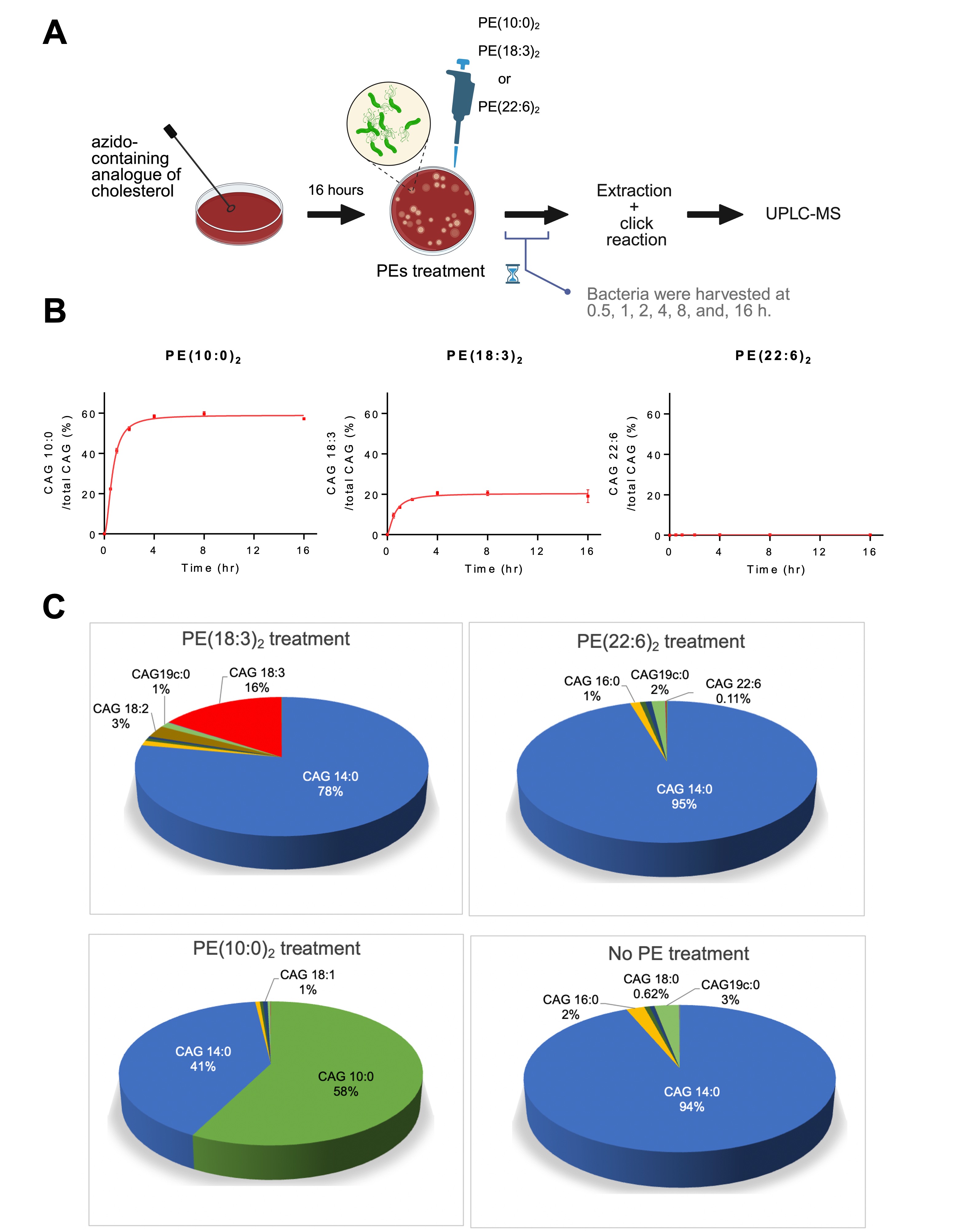

Supplement: Supplementary file 5 — Additional file 5: Supplemental Figure S5. Quantitative analysis of theconversion of PEs to corresponding CAGs in H. pylori.(A) A flowchart of measuring the conversion of PEs to corresponding CAGs in H. pylori. 17β-([3’-Azidopropoxy)-5-androsten-3β-ol (an azide-containing analogue of cholesterol) was added to H. pylori cells culture medium (at the final conc of 50 µM) and incorporated into the biosynthetic pathway to produce the analogues of CAGs and other cholesteryl glucoside derivatives. PE(10:0)2, PE(18:3)2 or PE(22:6)2 was also added to the bacterial culture for 0.5, 1, 2, 4, 8, and, 16 h. After harvest and Folch extraction, these compounds were further reacted with a fluorescent alkyne (4-N-methylamino-1,8-napthalimidopropyne) via Cu(I)-catalyzed 1,3-dipolar cycloaddition to obtain the fluorophore-conjugated products. The resulting products were analyzed by UPLC-MS. (B) Ratios of specific CAGs to total CAGs were determined by analyzing the area of product ion signals in the UPLC-MS spectra. The levels of the incorporated CAGs (CAG10:0, CAG18:3, or CAG22:6) relative to total CAGs were measured at 0.5, 1, 2, 4, 8, and 16 h after treatment with PE(10:0)2, PE(18:3)2 or PE(22:6)2. The data were obtained from three biological replicates. (C) Pi-charts display percentages of various CAGs that were analyzed after H. pylori was treated with precursor PE(10:0)2, PE(18:3)2 or PE(22:6)2 for 16 h. [file 12929_2024_1031_MOESM5_ESM.jpg]

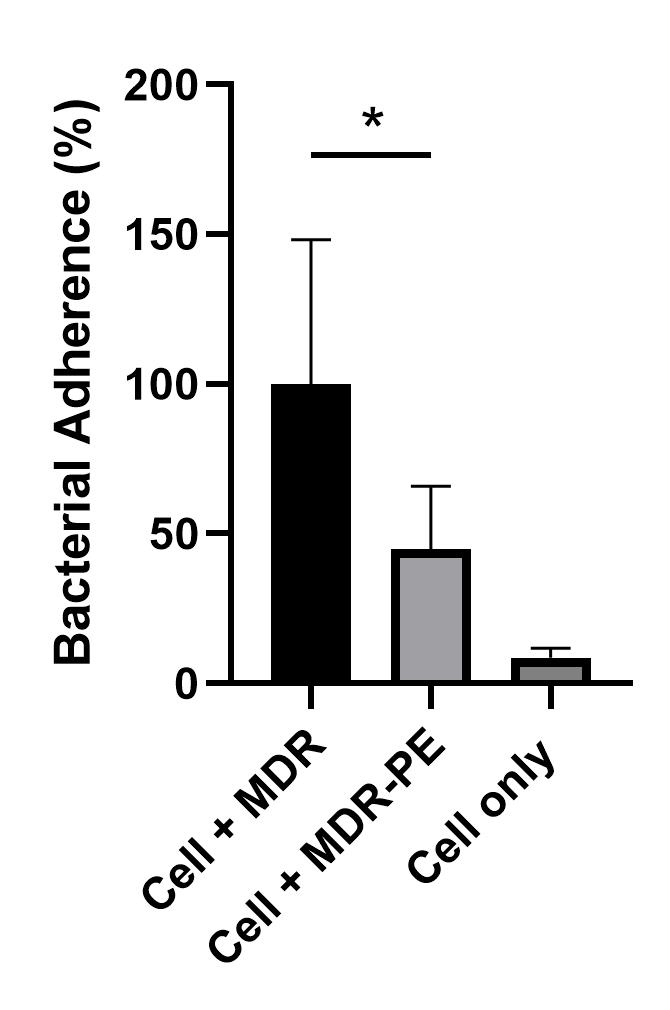

Supplement: Supplementary file 6 — Additional file 6: Supplemental Figure S6. Effect of PE(10:0)2 to inhibit the adhesion of the multidrug resistant H. pylori to AGS cells. The MDR strain of H. pylori MDR4955 was first treated with PE(10:0)2 for 1 h and then cocultured with AGS cells (MOI = 50) for another 1 h. The cells were detached from plates by using trypsin after washes with Dulbecco’s phosphate-buffered saline for three times, fixed with 2% formaldehyde, and then subjected to flow cytometry analysis. H. pylori-specific antibody (Abcam, ab20459, 1:1000) was used. Adherence was measured as the proportion of adhered AGS cells with H. pylori. The quantification of cell adhesion was normalized relative to the highest group, which was the "Cell with MDR infection," set as 100%. Representative data are shown as mean ± SD (standard error) (n = 6). Statistical analysis was performed using Student's t test with p-values less than 0.05 shown as one asterisk. Cell + MDR, cell infected by MDR4955; Cell + MDR-PE, cell infected by PE(10:0)2 - treated MDR4955. [file 12929_2024_1031_MOESM6_ESM.jpg]
