# Supplementary material for: Membrane lipid remodeling eradicates Helicobacter pylori by manipulating the cholesteryl 6'-acylglucoside biosynthesis
Source: J Biomed Sci. 2024 Apr 29;31:44. doi: 10.1186/s12929-024-01031-8 (PMC11057186; doi:10.1186/s12929-024-01031-8)
Supplement: Supplementary file 7 — Additional file 7: Supplemental Figure S7. A proposed model to explain how the acyl chain affects membrane dynamics. A proposed model explaining how CAGs containing different acyl chains (e.g., 18:0, 10:0, 18:3, 22:6) remodel the cell membrane to either enhance or reduce bacterial adhesion. Created with BioRender.com. [file 12929_2024_1031_MOESM7_ESM.pdf]

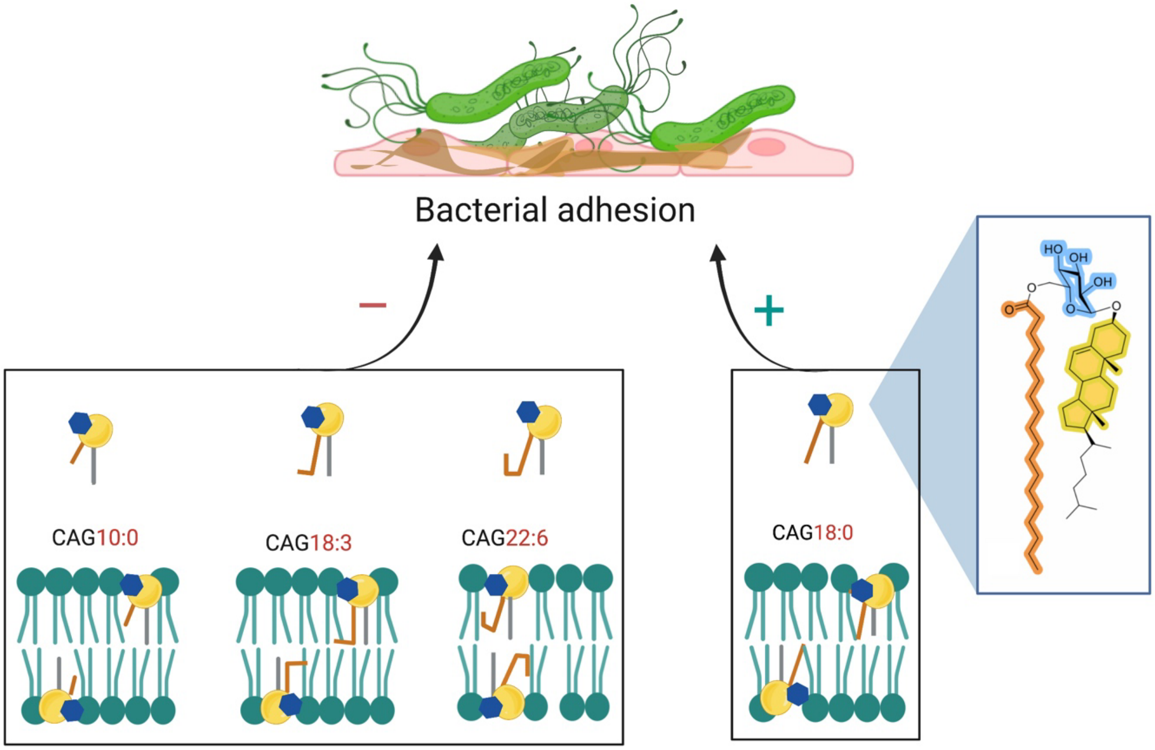

Supplemental Figure S7. A proposed model to explain how the acyl chain affects membrane dynamics. A proposed model explaining how CAGs containing different acyl chains (e.g., 18:0, 10:0, 18:3, 22:6) remodel the cell membrane to either enhance or reduce bacterial adhesion. Created with BioRender.com
