# Supplementary material for: Membrane lipid remodeling eradicates Helicobacter pylori by manipulating the cholesteryl 6'-acylglucoside biosynthesis
Source: J Biomed Sci. 2024 Apr 29;31:44. doi: 10.1186/s12929-024-01031-8 (PMC11057186; doi:10.1186/s12929-024-01031-8)
Supplement: Supplementary file 8 — Additional file 8: Supplemental Figure S8. Comparison of PE(10:0)2 and amiodarone in the bacterial adhesion.One multi-drug resistant strain of H. pylori MDR4955 was treated with PE(10:0)2 (100 µM) or amiodarone (50 µM) for 1 h and then cocultured with AGS cells (MOI = 50) for another 1 h. The resulting cells were detached from plates by using trypsin, washed with Dulbecco’s phosphate-buffered saline for three times, fixed with 2% formaldehyde, and subsequently subjected to flow cytometry analysis. H. pylori-specific antibody (Abcam, ab20459, 1:1000) was used for detecting the bacterial adhesion. The degree of cell adhesion was normalized relative to the highest group (AGS cells infected with MDR4955), set as 100%. Adherence was shown as the proportion of adhered AGS cells with H. pylori. Representative data are shown as mean ± SD (standard deviation) (n=3 for the groups treated with PE(10:0)2 and n=2 for the groups treated with amiodarone). Statistical analysis was performed using unpaired t test with Welch’s correction. p-Value is less than 0.05 shown as one asterisk, and less than 0.01 shown as two asterisks. Abbreviations: Cell + MDR, AGS cells cocultured with MDR4955; Cell + MDR + Ami, AGS cells cocultured with MDR4955 that were pretreated with amiodarone; Cell + MDR + PE10, AGS cells cocultured with MDR4955 that were pretreated with PE(10:0)2. [file 12929_2024_1031_MOESM8_ESM.pdf]

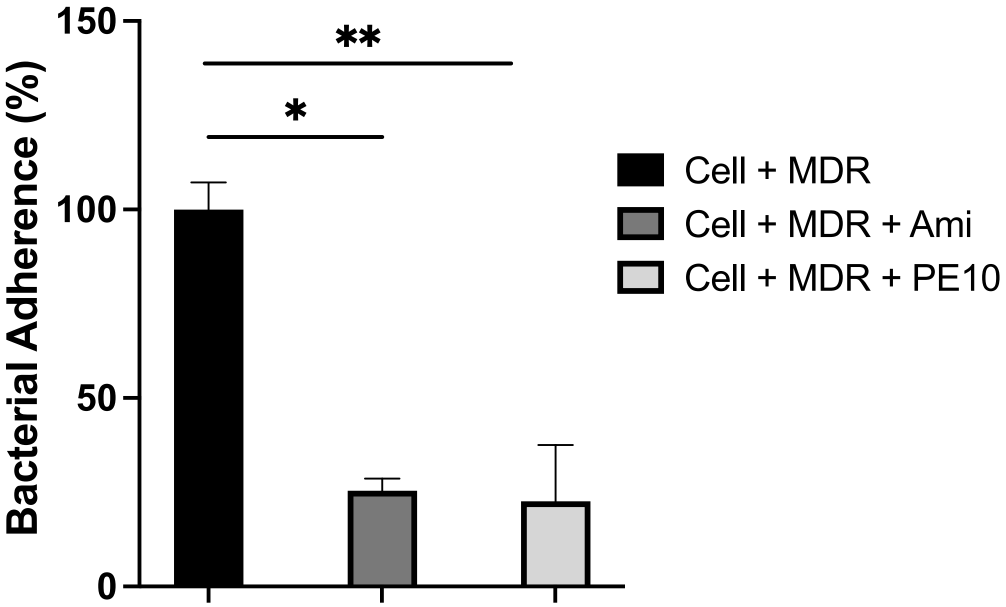

Supplemental Figure S8. Comparison of $PE(10:0)_2$ and amiodarone in the bacterial adhesion. One multi-drug resistant strain of *H. pylori* MDR4955 was treated with $PE(10:0)_2$ (100 μM) or amiodarone (50 μM) for 1 h and then cocultured with AGS cells (MOI = 50) for another 1 h. The resulting cells were detached from plates by using trypsin, washed with Dulbecco's phosphate-buffered saline for three times, fixed with 2% formaldehyde, and subsequently subjected to flow cytometry analysis. *H. pylori*-specific antibody (Abcam, ab20459, 1:1000) was used for detecting the bacterial adhesion. The degree of cell adhesion was normalized relative to the highest group (AGS cells infected with MDR4955), set as 100%. Adherence was shown as the proportion of adhered AGS cells with *H. pylori*. Representative data are shown as mean ± SD (standard deviation) (n=3 for the groups treated with $PE(10:0)_2$ and n=2 for the groups treated with amiodarone). Statistical analysis was performed using unpaired t test with Welch's correction. p-Value is less than 0.05 shown as one asterisk, and less than 0.01 shown as two asterisks. Abbreviations: Cell + MDR, AGS cells cocultured with MDR4955; Cell + MDR + Ami, AGS cells cocultured with MDR4955 that were pretreated with amiodarone; Cell + MDR + PE10, AGS cells cocultured with MDR4955 that were pretreated with $PE(10:0)_2$.
