# Supplementary material for: Membrane lipid remodeling eradicates Helicobacter pylori by manipulating the cholesteryl 6'-acylglucoside biosynthesis
Source: J Biomed Sci. 2024 Apr 29;31:44. doi: 10.1186/s12929-024-01031-8 (PMC11057186; doi:10.1186/s12929-024-01031-8)
Supplement: Supplementary file 9 — Additional file 9: Supplemental Figure S9. Effect of PE(10:0)2 or amiodarone on the OMVs secreted. The cells of H. pylori 26695 were treated with either PE(10:0)2 (100 µM; results shown by red color) or amiodarone (50 µM; results by green color) for 3 days. The level of secreted OMVs from H. pylori were monitored at 24 h, 48 h and 72 h. Secreted OMVs were isolated according to previous procedure, followed by labelling with a fluorescent lipophilic dye, FM4-64. The OMV level was quantified by detecting the corresponding excitation/emission at 515/640 nm and then normalized on the basis of cell number. Representative data are shown as mean ± SD (standard deviation) (n ≥ 2). Abbreviation: HP only, H. pylori with no treatment; HP + PE, H. pylori treated with PE(10:0)2; HP + Ami, H. pylori treated with amiodarone. [file 12929_2024_1031_MOESM9_ESM.pdf]

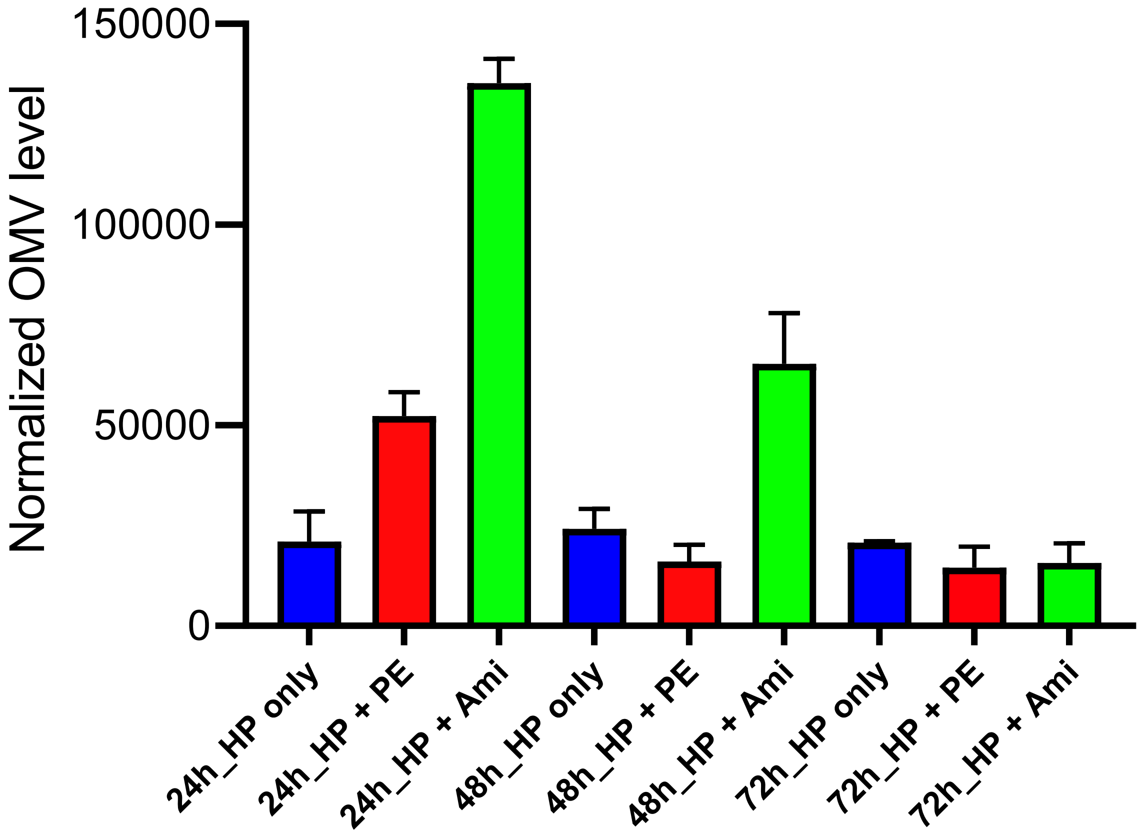

Supplemental Figure S9. Effect of $PE(10{:}0)_2$ or amiodarone on the OMVs secreted. The cells of *H. pylori* 26695 were treated with either $PE(10{:}0)_2$ (100 µM; results shown by red color) or amiodarone (50 µM; results by green color) for 3 days. The level of secreted OMVs from *H. pylori* were monitored at 24 h, 48 h and 72 h. Secreted OMVs were isolated according to previous procedure, followed by labelling with a fluorescent lipophilic dye, FM4-64. The OMV level was quantified by detecting the corresponding excitation/emission at 515/640 nm and then normalized on the basis of cell number. Representative data are shown as mean ± SD (standard deviation) ($n \geqslant 2$). Abbreviation: HP only, *H. pylori* with no treatment; HP + PE, *H. pylori* treated with PE(10:0)2; HP + Ami, *H. pylori* treated with amiodarone.
