# Supplementary material for: Membrane lipid remodeling eradicates Helicobacter pylori by manipulating the cholesteryl 6'-acylglucoside biosynthesis
Source: J Biomed Sci. 2024 Apr 29;31:44. doi: 10.1186/s12929-024-01031-8 (PMC11057186; doi:10.1186/s12929-024-01031-8)
Supplement: Supplementary file 10 — Additional file 10: Supplemental Figure S10. The cells of H. pylori 26695 were treated with either PE(10:0)2 (100 µM) or amiodarone (50 µM), followed by isolating the secreted OMVs to detect the VacA level at 24 h, 48 h and 72 h.(A) Western blot analysis of VacA protein. Abbreviations: H, H. pylori with no treatment; P, H. pylori treated with PE(10:0)2; A, H. pylori treated with amiodarone. ‘No-stain’ labelling was used as the loading control for the purpose of quantification. The quantification of VacA levels was normalized relative to the first 24 h of H. pylori control group, set as 100%. (B) Relative VacA level was measured by quantifying the density of each band and normalized according to the density of the corresponding total proteins (namely ‘No-stain’ labelling). Summarized quantification data from independent western blots (n=4) are shown as mean ± S.E.M. (standard error of the mean). **** P < 0.0001 vs. control, *** P < 0.001 vs. control, ** P < 0.01 vs. control (H. pylori only in each group at 24, 48 or 72 h). Statistical analyses were performed using two-way analysis of variance (ANOVA). [file 12929_2024_1031_MOESM10_ESM.pdf]

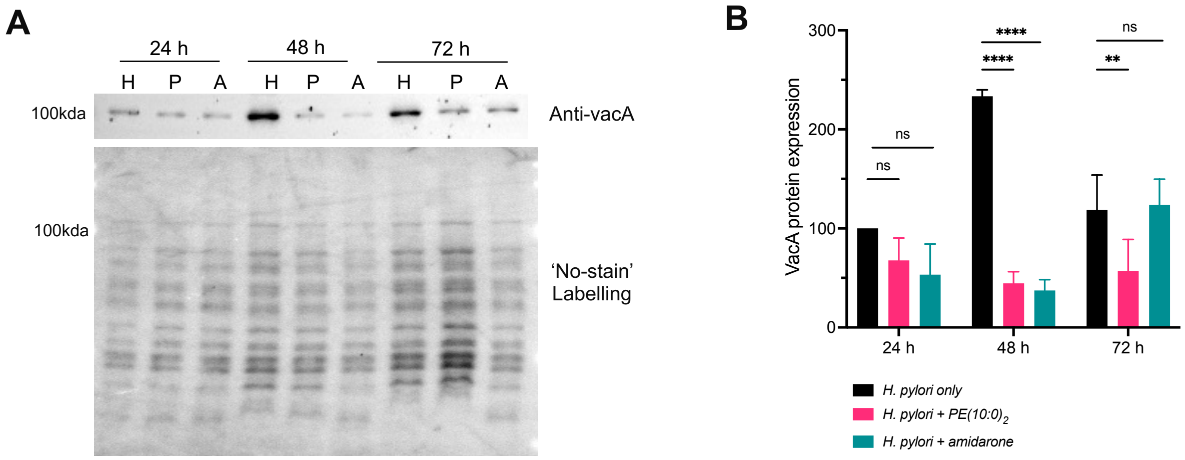

Supplemental Figure S10. The cells of *H. pylori* 26695 were treated with either PE(10:0)$_2$ (100 μM) or amiodarone (50 μM), followed by isolating the secreted OMVs to detect the VacA level at 24 h, 48 h and 72 h. (A) Western blot analysis of VacA protein. Abbreviations: H, *H. pylori* with no treatment; P, *H. pylori* treated with PE(10:0)$_2$; A, *H. pylori* treated with amiodarone. 'No-stain' labelling was used as the loading control for the purpose of quantification. The quantification of VacA levels was normalized relative to the first 24 h of *H. pylori* control group, set as 100%. **(B)** Relative VacA level was measured by quantifying the density of each band and normalized according to the density of the corresponding total proteins (namely 'No-stain' labelling). Summarized quantification data from independent western blots (n=4) are shown as mean ± S.E.M. (standard error of the mean). **** $P < 0.0001$ vs. control, *** $P < 0.001$ vs. control, ** $P < 0.01$ vs. control (*H. pylori* only in each group at 24, 48 or 72 h). Statistical analyses were performed using two-way analysis of variance (ANOVA).
