# Supplementary material for: Membrane lipid remodeling eradicates Helicobacter pylori by manipulating the cholesteryl 6'-acylglucoside biosynthesis
Source: J Biomed Sci. 2024 Apr 29;31:44. doi: 10.1186/s12929-024-01031-8 (PMC11057186; doi:10.1186/s12929-024-01031-8)
Supplement: Supplementary file 11 — Additional file 11. 1H and 13C NMR spectra of CAGs (see Appendixes) [file 12929_2024_1031_MOESM11_ESM.docx]

**Cholesteryl 6-*O*-decanoyl-*α*-glucoside** (**CAG 10:0**)

For **CAG 10:0**, [*α*]_D_^30^ = +88.0 (*c* = 1.0, CHCl_3_); ^1^H NMR (400 MHz, CDCl_3_): δ = 5.35 (d, *J* = 5.2 Hz, 1H), 5.02 (d, *J* = 4.0 Hz, 1H), 4.51 (dd, *J* = 12.4, 4.8 Hz, 1H), 4.23 (dd, *J* = 12.0, 2.0 Hz, 1H), 3.90-3.81 (m, 1H), 3.72 (t, *J* = 9.6 Hz, 1H), 3.53-3.42 (m, 2H), 3.33 (t, *J* = 9.6 Hz, 1H), 3.01 (s, 1H), 2.76 (s, 1H), 2.41-2.27 (m, 4H), 2.11-1.75 (m, 6H), 1.68-1.21 (m, 28H), 1.20-0.96 (m, 11H), 0.94-0.81 (m, 12H), 0.68 (s, 3H). 13C NMR (75 MHz, CDCl_3_) δ = 174.2, 140.4, 122.1, 96.8, 78.3, 74.4, 71.9, 70.4, 69.9, 63.6, 56.7, 56.2, 50.1, 42.3, 40.1, 39.7, 39.5, 37.0, 36.6, 36.2, 35.8, 34.3, 31.9, 31.8, 29.6, 29.4, 28.2, 28.0, 24.9, 24.3, 23.8, 22.8, 22.7, 22.5, 21.1, 19.3, 18.7, 14.1, 11.8. HRMS (ESI): calcd. for C_43_H_74_O_7_[M + Na]^+^, 725.5327; found 725.5326.

**Cholesteryl 6-*O*-myristoyl-*α*-glucoside** (**CAG** **14:0**)

For **CAG** **14:0**, [*α*]_D_^30^ = +28.0 (*c* = 0.5, CHCl_3_); ^1^H NMR (300 MHz, CDCl_3_): δ = 5.34 (d, *J* = 4.8 Hz, 1H), 4.99 (d, *J* = 4.0 Hz, 1H), 4.38 (dd, *J* = 12.0, 5.2Hz, 1H), 4.34-4.28 (m, 1H), 3.98-3.82 (m, 2H), 3.72 (t, *J* = 9.2 Hz, 2H), 3.53-3.42 (m, 1H), 3.33 (t, *J* = 9.6 Hz, 1H), 2.82 (s, 1H), 2.41-2.27 (m, 4H), 2.09-1.78 (m, 6H), 1.67-1.21 (m, 33H), 1.19-0.95 (m, 12H), 0.94-0.81 (m, 12H), 0.68 (s, 3H).^13^C NMR (100 MHz, CDCl_3_) δ = 174.4, 140.3, 122.1, 96.8, 78.4, 74.5, 72.0, 70.2, 69.9, 63.4, 56.7, 56.2, 50.1, 42.3, 40.1, 39.7, 39.5, 37.0, 36.6, 36.2, 35.8, 34.3, 31.9, 31.9, 29.7, 29.7, 29.7, 29.7, 29.6, 29.4, 29.4, 29.3, 28.2, 28.0, 24.9, 24.3, 23.8 22.8, 22.7, 22.5, 21.0, 19.3, 18.7, 14.1, 11.8. HRMS (ESI): calcd. for C_47_H_82_O_7_[M + Na]^+^, 781.5993; found 781.5951.

**Cholesteryl 6-*O*-palmitoyl-*α*-glucoside (CAG 16:0**)

For **CAG 16:0**, *R*_f_ 0.14 (CH_2_Cl_2_/MeOH 5/0.4); [*α*]_D_^30^ = +27 (*c* 1.03, CHCl_3_); ^1^H NMR (500 MHz, CDCl_3_) δ 5.33 (s, 1H), 4.97 (d, *J* = 2.5 Hz, 1H, H-1′), 4.66 (s, 1H), 4.36 (d, *J* = 11.0 Hz, 1H), 4.30 – 4.27 (m, 2H), 3.87 (t, *J* = 6.5 Hz, 1H), 3.73 (t, *J* = 9.0 Hz, 1H), 3.48 (s, 2H), 3.33 (t, *J* = 9.0 Hz, 1H), 2.35 – 2.31 (m, 4H), 2.02 – 1.83 (m, 5H), 1.60 – 1.49 (m, 7H), 1.33 – 1.25 (m, 31H), 1.15 – 1.00 (m, 12H), 0.92 – 0.86 (m, 12H), 0.68 (s, 3H); ^13^C NMR (125 MHz, CDCl_3_) δ 174.3, 140.5, 122.1, 97.0, 78.4, 74.5, 72.0, 70.5, 70.0, 63.8, 56.8, 56.3, 50.2, 42.4, 40.2, 39.9, 39.6, 37.2, 36.7, 36.3, 35.9, 34.4, 32.03, 31.96, 29.92, 29.88, 29.86, 29.8, 29.53, 29.48, 28.3, 28.1, 28.0, 25.1, 24.4, 24.0, 22.9, 22.8, 22.6, 21.2, 19.5, 18.8, 14.2, 11.9; HRMS-ESI (*m/z*): [M + Na]^+^ calcd for C_49_H_86_NaO_7_^+^, 809.6266; found, 809.6259.

**Cholesteryl 6-*O*-stearoyl-*α*-glucoside** (**CAG 18:0**)

For **CAG 18:0**, *R*_f_ 0.16 (hexanes/EtOAc 1/3); [*α*]_D_^30^= +34.0 (*c* 1.0, CHCl_3_); ^1^H NMR (500 MHz, CDCl_3_) δ 5.34 (s, 1H), 4.98 (d, *J* = 3.5 Hz, 1H, H-1′), 4.34 (s, 3H), 3.87 (s, 1H), 3.73 (t, *J* = 9.0 Hz, 1H), 3.48 (s, 2H), 3.33 (t, *J* = 9.0 Hz, 1H), 2.34 (d, *J* = 8.0 Hz, 4H), 2.02 – 1.84 (m, 5H), 1.61 – 1.49 (m, 8H), 1.34 – 1.26 (m, 32H), 1.15 – 1.00 (m, 13H), 0.92 – 0.86 (m, 13H), 0.68 (s, 3H); ^13^C NMR (125 MHz, CDCl_3_) δ 174.4, 140.6, 122.2, 97.1, 78.6, 74.6, 72.1, 70.5, 70.1, 63.7, 56.9, 56.4, 50.3, 42.5, 40.3, 39.9, 39.7, 37.2, 36.8, 36.4, 36.0, 34.5, 32.1, 32.1, 30.0, 29.9, 29.59, 29.55, 28.4, 28.2, 28.1, 25.1, 24.5, 24.1, 23.0, 22.9, 22.7, 21.3, 19.54, 18.9, 14.3, 12.0; HRMS-ESI (*m/z*): [M + Na]^+^ calculated for C_51_H_90_NaO_7_^+^, 837.6579; found, 837.6582.

**Cholesteryl 6-*O*-Arachidoyl-*α*-glucoside** (**CAG 20:0**)

For **CAG 20:0**, *R*_f_ 0.14 (CH_2_Cl_2_/EtOAc 1/1); [*α*]_D_^30^ = +36.0 (*c* 0.9, CHCl_3_); ^1^H NMR (500 MHz, CDCl_3_) δ 5.34 (d, *J* = 5.0 Hz, 1H), 5.00 (d, *J* = 4 Hz, 1H, H-1′), 4.42 (dd, *J* = 12.0, 5.0 Hz, 1H), 4.30 (dd, *J* = 12.0, 2.0 Hz, 1H), 3.85 – 3.88 (m, 1H), 3.72 (t, *J* = 9.0 Hz, 1H), 3.56 (d, *J* = 23.0 Hz, 2H), 3.50 – 3.45 (m, 2H), 3.33 (t, *J* = 10.5 Hz, 1H), 2.35 (t, *J* = 7.5 Hz, 3H), 2.02 – 1.87 (m, 7H), 1.63 – 1.48 (m, 7H), 1.35 – 1.25 (m, 37H), 1.16 – 0.98 (m, 12H), 0.92 – 0.86 (m, 13H), 0.68 (s, 3H); ^13^C NMR (125 MHz, CDCl_3_) δ 174.6, 140.4, 122.3, 97.0 (C-1′, *J*_CH′_ = 168.125 Hz), 78.5, 74.6, 72.1, 70.3, 70.1, 63.4, 56.8, 56.3, 50.2, 42.4, 40.2, 39.8, 39.6, 37.1, 36.7, 36.3, 35.9, 34.4, 32.02, 31.96, 29.83, 29.81, 29.76, 29.72, 29.5, 29.43, 29.37, 28.3, 28.1, 25.1, 24.4, 24.0, 22.9, 22.8, 22.6, 21.2, 19.4, 18.8, 14.2, 11.9; HRMS-ESI (*m/z*): [M + Na]^+^ calculated for C_53_H_94_NaO_7_^+^, 865.6892; found, 865.6889.

**Cholesteryl 6-*O*-Behenoyl-*α*-glucoside (CAG 22:0)**

For **CAG 22:0**, *R*_f_ 0.11 (hexanes/EtOAc 1/2); [*α*]_D_^30^ = +34.22 (*c* 1.23, CHCl_3_); ^1^H NMR (500 MHz, CDCl_3_) δ 5.33 (s, 1H), 4.97 (d, *J* = 2.5 Hz, 1H, H-1′), 4.36 (d, *J* = 11.0 Hz, 1H), 4.27 (dd, *J* = 12.0, 6.5 Hz, 1H), 3.88 (t, *J* = 8.0 Hz, 1H), 3.72 (t, *J* = 9.0 Hz, 1H), 3.43 – 3.49 (m, 2H), 3.32 (t, *J* = 9.5 Hz, 1H), 2.35 – 2.30 (m, 3H), 2.02 – 1.83 (m, 4H), 1.61 – 1.42 (m, 9H), 1.38 – 1.26 (m, 42H), 1.16 – 0.98 (m, 12H), 0.92 – 0.86 (m, 13H), 0.68 (s, 3H); ^13^C NMR (125 MHz, CDCl_3_) δ 174.3, 140.6, 122.1, 97.0 (C-1′, *J*_CH′_ = 168.125 Hz), 78.5, 74.4, 72.0, 70.6, 70.0, 63.8, 56.8, 56.3, 50.2, 42.4, 40.2, 39.9, 39.6, 37.2, 36.7, 36.3, 35.9, 34.4, 32.02, 31.97, 30.0, 29.91, 29.88, 29.87, 29.85, 29.84, 29.81, 29.76, 29.6, 29.50, 29.46, 28.3, 28.1, 28.0, 25.0, 24.4, 24.0, 22.9, 22.8, 22.6, 21.2, 19.5, 18.8, 14.2, 11.9; HRMS-ESI (m/z): [M + H]^+^ calculated for C_55_H_99_O_7_^+^, 871.7385; found, 871.7368.

**Cholesteryl 6-*O*-oleoyl-*α*-glucoside (CAG 18:1)**

For **CAG 18:1**, *R*_f_ 0.10 (hexanes/EtOAc 1/1); [*α*]_D_^30^ = +34.0 (*c* 0.65, CHCl_3_); ^1^H NMR (400 MHz, CDCl_3_) δ 5.35 – 5.32 (m, 2H), 4.99 (d, *J* = 3.9 Hz, 1H), 4.38 (dd, *J* = 12.4, 5.6 Hz, 1H), 4.31 (dd, *J* = 12.0, 1.6 Hz, 1H), 3.90 – 3.84 (m, 2H), 3.75 – 3.70 (m, 2H), 3.48 (td, *J* = 9.6, 4.0 Hz, 2H), 3.32 (td, *J* = 9.6, 3.2 Hz, 1H), 2.79 (d, *J* = 9.6 Hz, 1H), 2.36 – 2.32 (m, 4H), 2.04 – 1.98 (m, 7H), 1.94 – 1.78 (m, 3H), 1.62 – 1.42 (m, 9H), 1.34 – 1.24 (m, 24H), 1.19 – 1.04 (m, 6H), 1.02 – 0.97 (m, 6H), 0.92 – 0.85 (m, 13H), 0.67 (s, 3H); ^13^C NMR (125 MHz, CDCl_3_) δ 174.6, 140.6, 130.2, 129.9, 122.3, 97.1, 78.7, 74.8, 72.2, 70.5, 70.2, 63.6, 57.0, 56.4, 50.4, 42.5, 40.3, 40.0, 39.7, 37.3, 36.9, 36.4, 36.0, 34.5, 32.2, 32.13, 32.10, 30.0, 29.8, 29.56, 29.55, 29.48, 28.5, 28.2, 27.5, 25.2, 24.5, 24.1, 23.0, 22.9, 22.8, 21.3, 19.6, 18.9, 14.3, 12.1;HRMS-ESI (*m/z*): [M + Na]^+^ calculated for C_51_H_89_O_7_^+^, 813.6603; found, 813.6603.

**Cholesteryl 6-*O*-Linoleoyl-*α*-glucoside (CAG 18:2)**

For **CAG 18:2**, *R*_f_ 0.17 (CH_2_Cl_2_/EtOAc 1/1); [*α*]_D_^30^ = +37.8 (*c* 0.37, CHCl_3_); ^1^H NMR (500 MHz, CDCl_3_) δ 5.40 – 5.32 (m, 5H) , 5.01 (d, *J* = 4.0 Hz, 1H, H-1′), 4.45 (dd, *J* = 12.0, 5.0 Hz, 1H), 4.27 (d, *J* = 10.5 Hz, 1H), 3.86 (dd, *J* = 9.5, 3.0 Hz, 1H), 3.75 – 3.64 (m, 2H), 3.51 – 3.31 (m, 5H), 2.77 (t, *J* = 6.5 Hz, 2H), 2.35 (t, *J* = 7.5 Hz, 3H), 2.07 – 1.79 (m, 11H), 1.63 – 1.43 (m, 10H), 1.37 – 1.26 (m, 21H), 1.19 – 1.05 (m, 7H), 1.03 – 0.97 (m, 5H), 0.92 – 0.86 (m, 14H), 0.68 (s, 3H); ^13^C NMR (125 MHz, CDCl_3_) δ 174.6, 140.4, 130.3, 130.1, 128.2, 128.0, 122.3, 97.0 (C-1′, *J*_CH′_ = 171.25 Hz), 78.6, 74.7, 72.1, 70.2, 70.1, 63.3, 56.8, 56.3, 50.2, 42.4, 40.2, 39.8, 39.6, 37.1, 36.7, 36.3, 35.9, 34.3, 32.02, 31.96, 31.6, 29.8, 29.7, 29.4, 29.3, 28.3, 28.1, 27.31, 27.29, 25.7, 25.0, 24.4, 23.9, 22.9, 22.7, 22.6, 21.1, 19.4, 18.8, 14.2, 11.9; HRMS-ESI (*m/z*): [M + Na]^+^ calculated for C_55_H_86_NaO_7_^+^, 833.6266; found, 833.6304.

**Cholesteryl 6-*O*-Linolenyl-*α*-glucoside (CAG 18:3)**

For **CAG 18:3**, *R*_f_ 0.22 (hexanes/EtOAc 1/2); [*α*]_D_^30^ = +36 (*c* 0.5, CHCl_3_); ^1^H NMR (500 MHz, CDCl_3_) δ 5.42 – 5.29 (m, 7H), 5.00 (d, *J* = 5.0 Hz, 1H, H-1′), 4.43 (dd, *J* = 12.0, 5.0 Hz, 1H), 4.28 (d, *J* = 10.5 Hz, 1H), 3.86 (dd, *J* = 9.5, 3.0 Hz, 1H), 3.72 (t, *J* = 9.5 Hz, 1H), 3.51 – 3.45 (m, 3H), 3.35 – 3.27 (m, 1H), 2.80 (t, *J* = 6.5 Hz, 4H), 2.35 (t, *J* = 7.5 Hz, 6H), 2.11 – 1.79 (m, 13H), 1.63 – 1.43 (m, 14H), 1.38 – 1.26 (m, 34H), 1.16 – 0.96 (m, 25H), 0.92 – 0.84 (m, 23H), 0.68 (s, 4H); ^13^C NMR (125 MHz, CDCl_3_) δ 174.6, 140.4, 132.0, 130.3, 128.4, 128.3, 127.9, 127.2, 122.3, 97.0(C-1′, *J*_CH′_ = 167.6 Hz), 78.6, 74.6, 72.1, 70.2, 70.1, 63.4, 56.8, 56.3, 50.2, 42.4, 40.2, 39.8, 39.6, 37.1, 36.7, 36.3, 35.9, 34.3, 32.01, 31.95, 29.8, 29.7, 29.5, 29.3, 28.3, 28.1, 27.3, 25.7, 25.6, 25.0, 24.4, 24.0, 22.9, 22.8, 22.6, 21.1, 20.6, 19.4, 18.8, 14.4, 14.2, 11.9; HRMS-ESI (*m/z*): [M + Na]^+^ calculated for C_51_H_85_O_7_^+^, 809.6290; found, 809.6302.

**Cholesteryl 6-*O*-Arachidonyl-*α*-glucoside (CAG 20:4)**

For **CAG 20:4**, *R*_f_ 0.28 (hexanes/EtOAc 1/3); [*α*]_D_^30^ = +39 (*c* 0.7, CHCl_3_); ^1^H NMR (500 MHz, CDCl_3_) δ 5.43 – 5.31 (m, 9H), 5.01 (d, *J* = 4.0 Hz, 1H, H-1′), 4.50 (dd, *J* =12.0, 4.5 Hz, 1H), 4.24 (dd, *J* = 12.0, 2.0 Hz, 1H), 3.85 (dd, *J* = 9.5, 2.0 Hz, 1H), 3.72 (t, *J* = 9.5 Hz, 1H), 3.50 – 3.46 (m, 2H), 3.36 (t, *J* = 9.5 Hz, 1H), 2.85 – 2.79 (m, 6H), 2.40 – 2.33 (m, 5H), 2.17 – 1.79 (m, 14H), 1.75 – 1.43 (m, 24H), 1.37 – 1.25 (m, 36H), 1.06 – 1.01 (m, 19H), 0.92 – 0.86 (m, 24H), 0.68 (s, 3H); ^13^C NMR (125 MHz, CDCl_3_) δ 174.5, 140.4, 130.6, 129.1, 128.9, 128.7, 128.4, 128.19, 127.9, 127.6, 122.4, 97.0, 78.6, 74.7, 72.2, 70.1, 70.0, 63.2, 56.8, 56.3, 50.2, 42.4, 40.2, 39.8, 39.6, 37.1, 36.8, 36.3, 35.9, 33.7, 32.02, 31.96, 31.6, 29.8, 29.4, 28.3, 28.13, 28.11, 27.3, 26.6, 25.74, 25.72, 24.8, 24.4, 23.9, 22.9, 22.67, 22.65, 21.1, 19.4, 18.8, 14.2, 12.0; HRMS-ESI (*m/z*): [M + Na]^+^ calculated for C_55_H_86_NaO_7_^+^, 857.6266; found, 857.6272.

**Cholesteryl 6-*O*-Docosahexenoyl-*α*-Glucoside 1k (CAG 22:6)**

For **CAG 22:6**, *R*_f_ 0.11 (hexanes/EtOAc 1/1); [*α*]_D_^30^ = +37 (*c* 0.76, CHCl_3_); ^1^H NMR (500 MHz, CDCl_3_) δ 5.38 – 5.30 (m, 13H), 5.00 (d, *J* = 4.0 Hz, 1H, H-1′), 4.47 (dd, *J* =12.0, 4.5 Hz, 1H), 4.27 (d, *J* = 12.5 Hz, 1H), 3.87 – 3.85 (m, 1H), 3.72 (t, *J* = 4.5 Hz, 1H), 3.49 – 3.45 (m, 2H), 3.34 (t, *J* = 9.5 Hz, 1H), 2.85 – 2.80 (m, 10H), 2.44 – 2.34 (m, 8H), 2.11 – 1.79 (m, 9H), 1.78 – 1.43 (m, 9H), 1.35 – 1.26 (m, 19H), 1.13 – 1.05 (m, 10H), 1.03 – 0.96 (m, 10H), 0.92 – 0.83 (m, 19H), 0.68 (s, 3H); ^13^C NMR (125 MHz, CDCl_3_) δ 173.9, 140.4, 132.1, 129.6, 128.7, 128.40, 128.36, 128.35, 128.18, 128.16, 128.1, 128.0, 127.8, 127.1, 122.3, 97.0(C-1′, *J*_CH′_ = 167.875 Hz), 78.5, 74.6, 72.1, 70.0, 63.4, 56.8, 56.3, 50.2, 42.4, 40.2, 39.9, 39.6, 37.1, 36.8, 36.3, 35.9, 34.1, 32.01, 31.95, 29.8, 28.3, 28.1, 25.73, 25.69, 25.6, 24.4, 23.9, 22.9, 22.8, 22.6, 21.1, 20.7, 19.4, 18.8, 14.4, 14.2, 11.9; HRMS-ESI (*m/z*): [M + H]^+^ calculated for C_55_H_87_7O_7_^+^, 859.6446; found, 859.6456.

**^1^H NMR of CAG 10:0 (CDCl_3_, 400 MHz)**


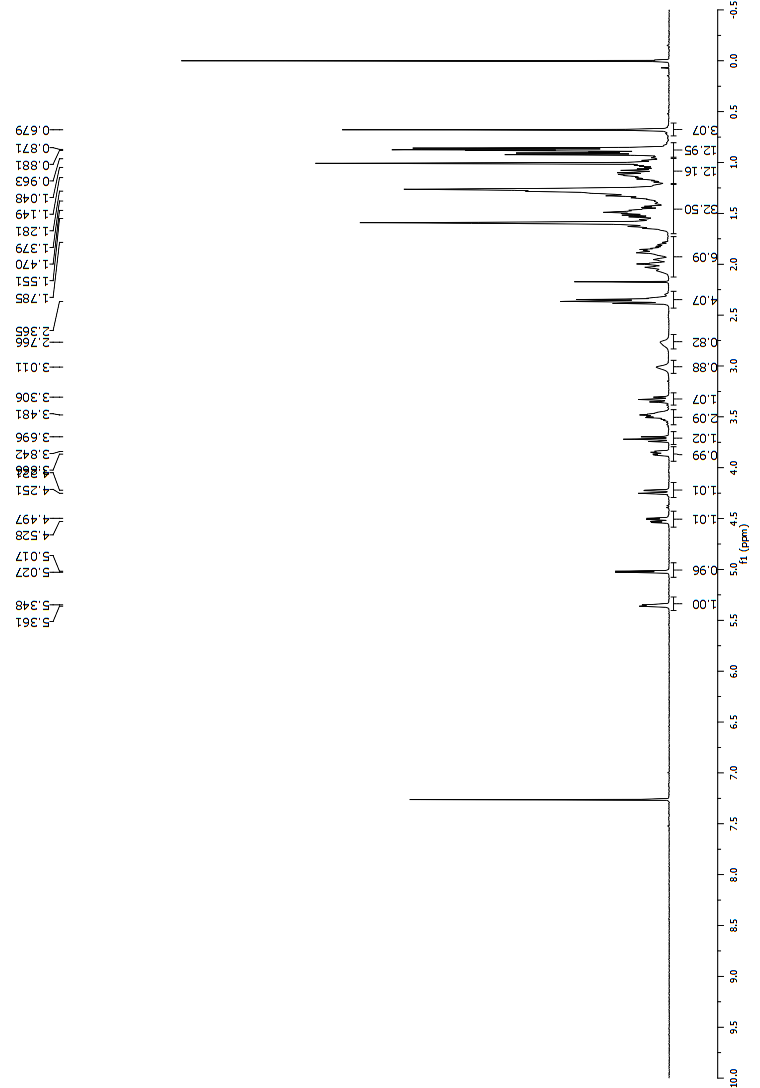


**^13^C NMR of CAG 10:0 (CDCl_3_, 100 MHz)**


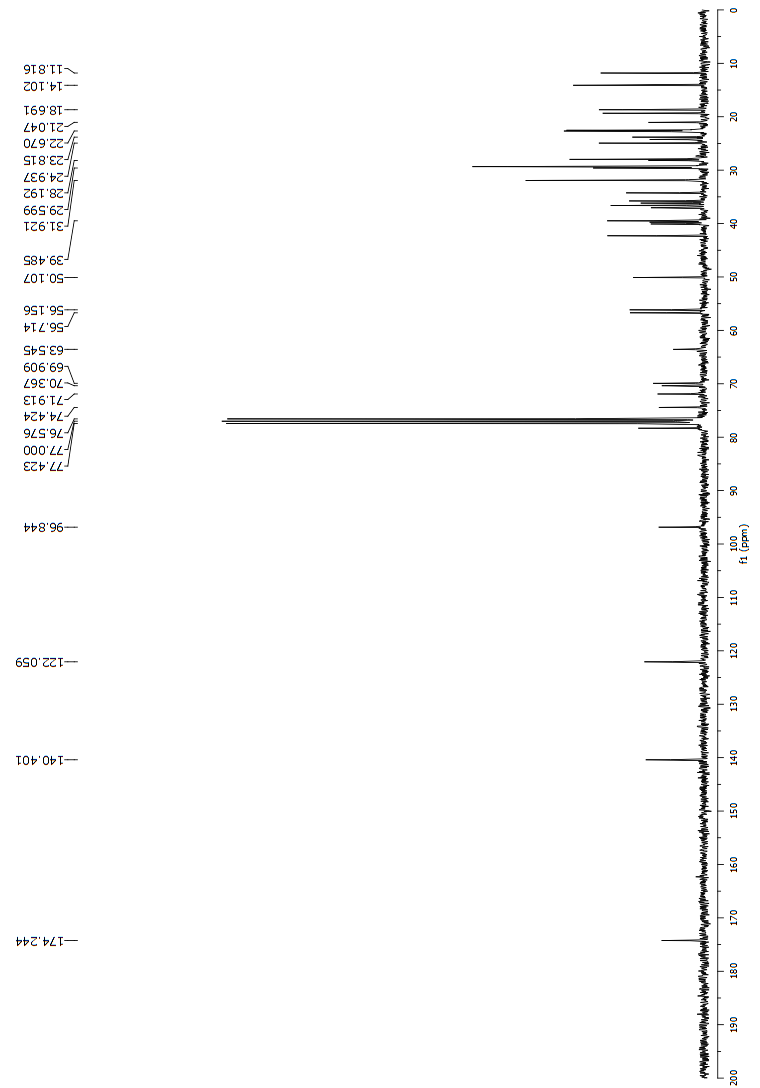


**^1^H NMR of CAG 14:0 (CDCl_3_, 400 MHz)**

^
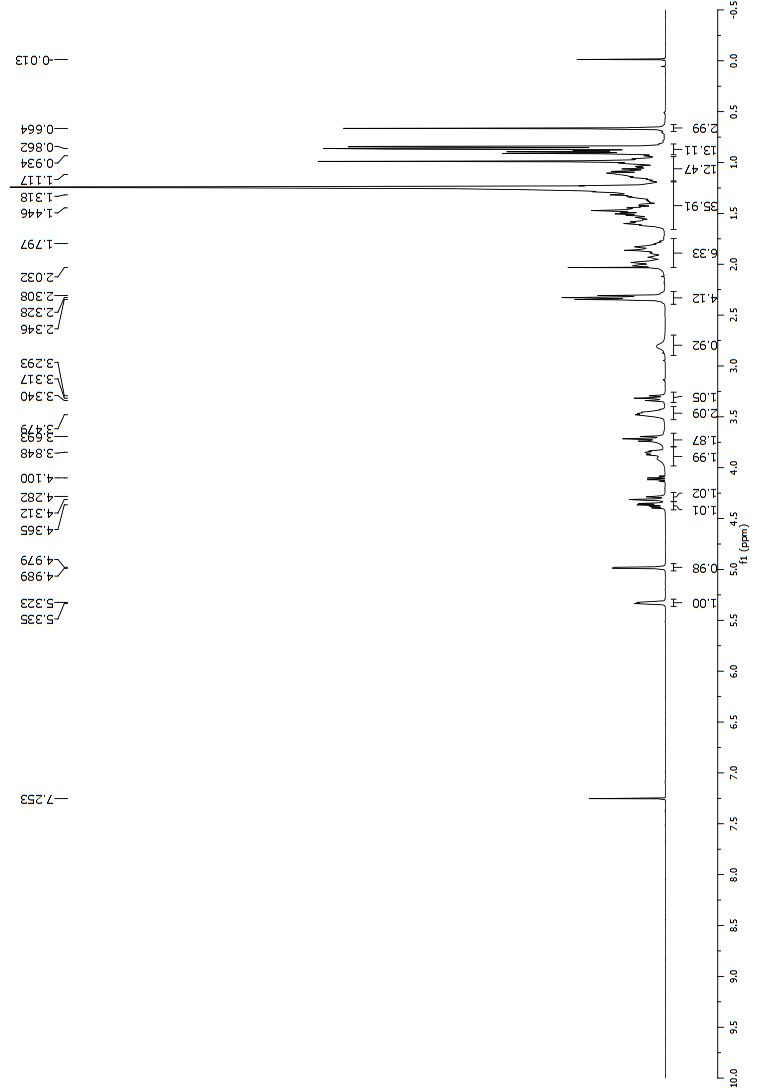
^

**^13^C NMR of CAG 14:0 (CDCl_3_, 100 MHz)**

^
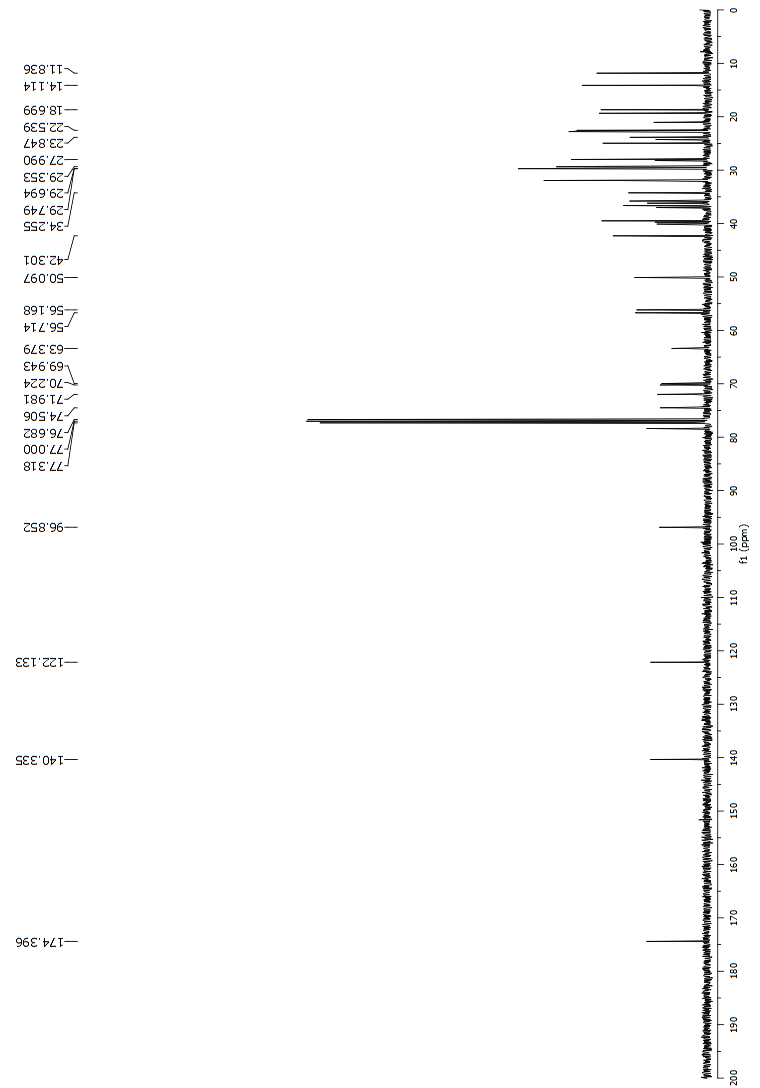
^

^1^H NMR spectrum of CAG 16:0 (CDCl_3_, 400 MHz)


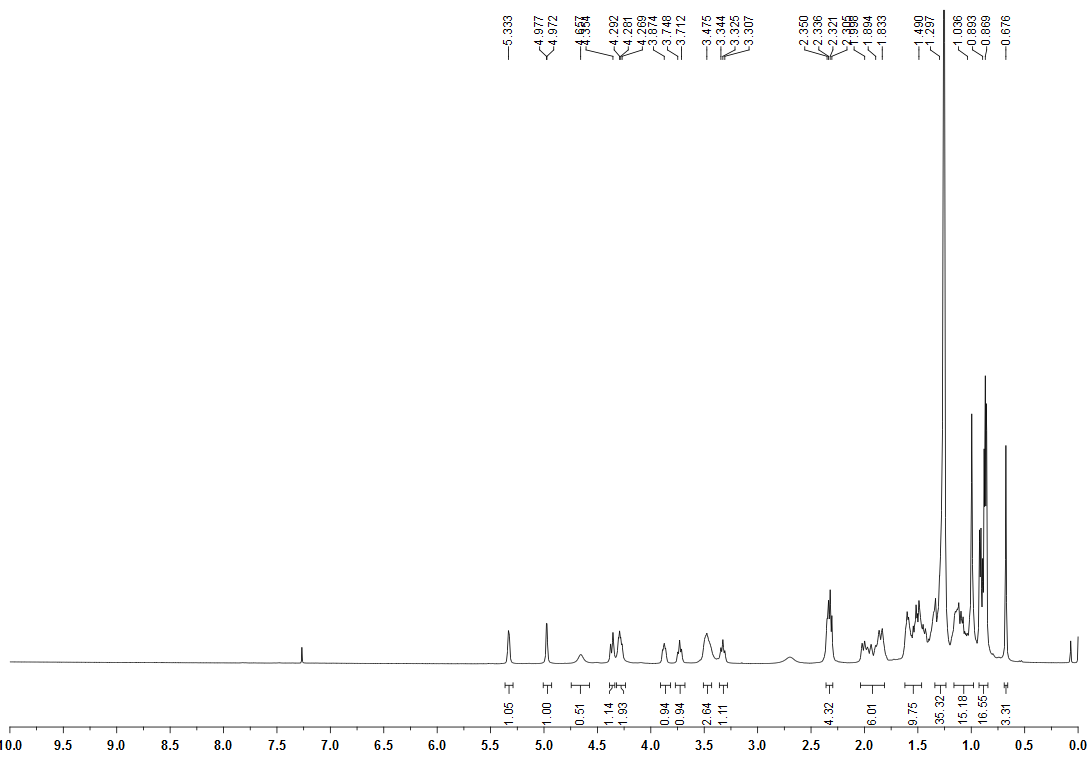


^13^C NMR spectrum of CAG 16:0 (CDCl_3_, 100 MHz)


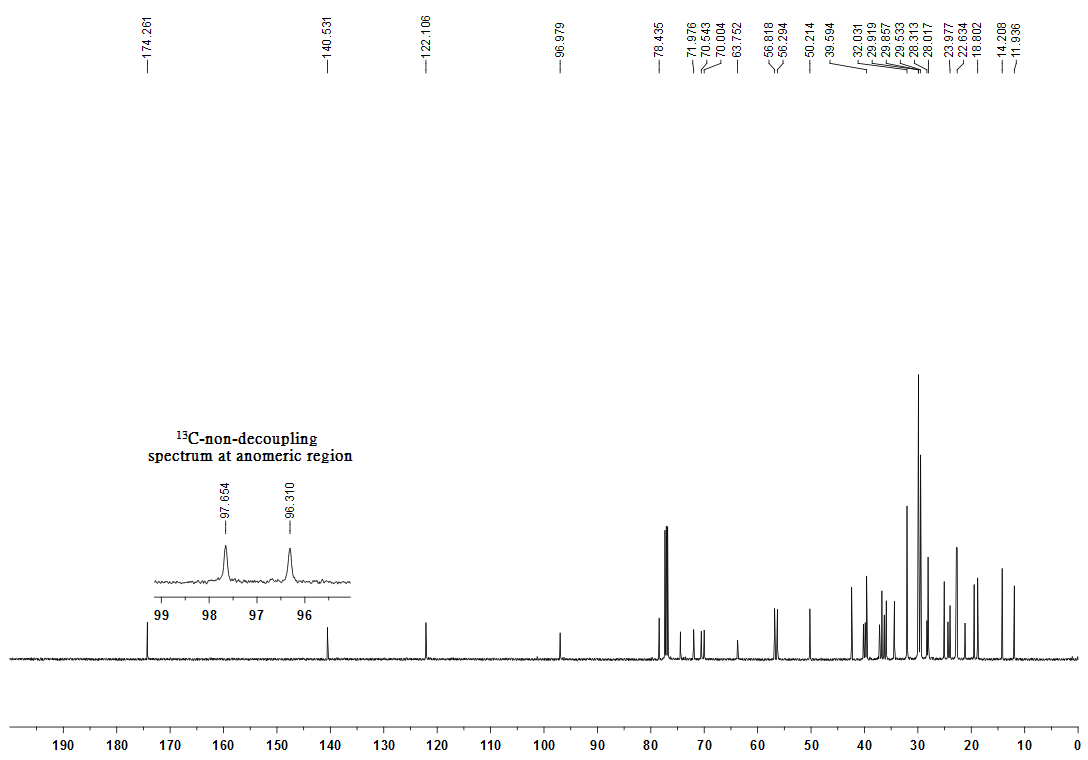


^1^H NMR spectrum of CAG 18:0 (CDCl_3_, 400 MHz)


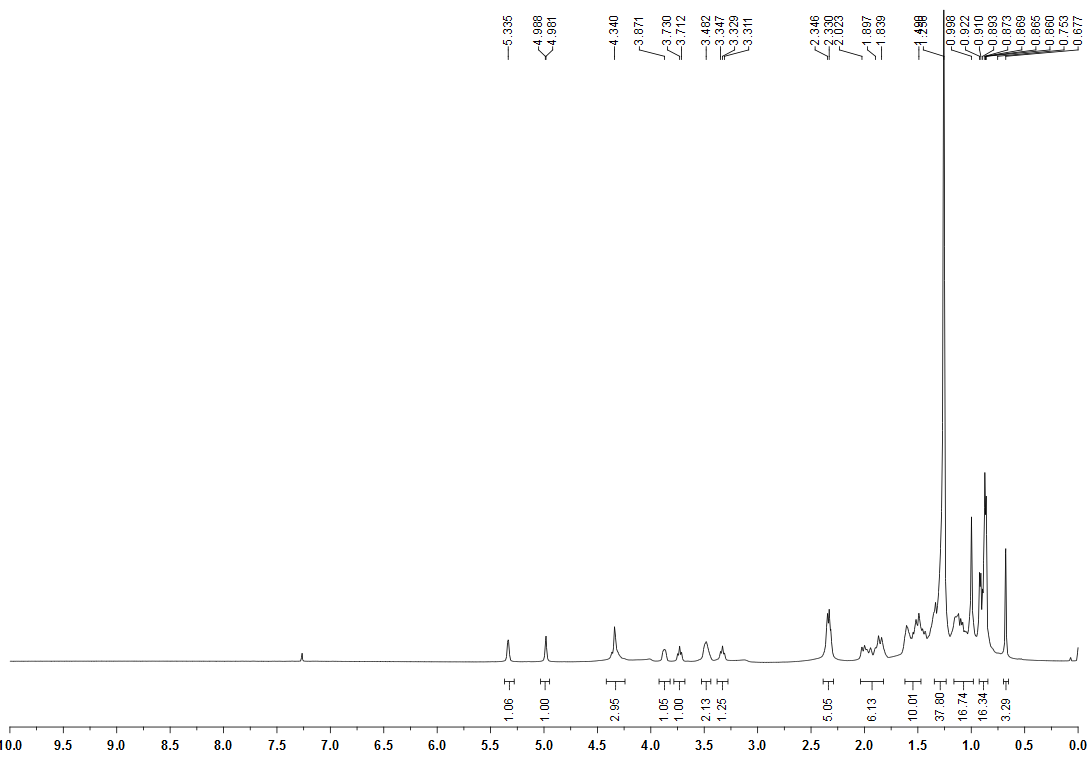


^13^C NMR spectrum of CAG 18:0 (CDCl_3_, 100 MHz)


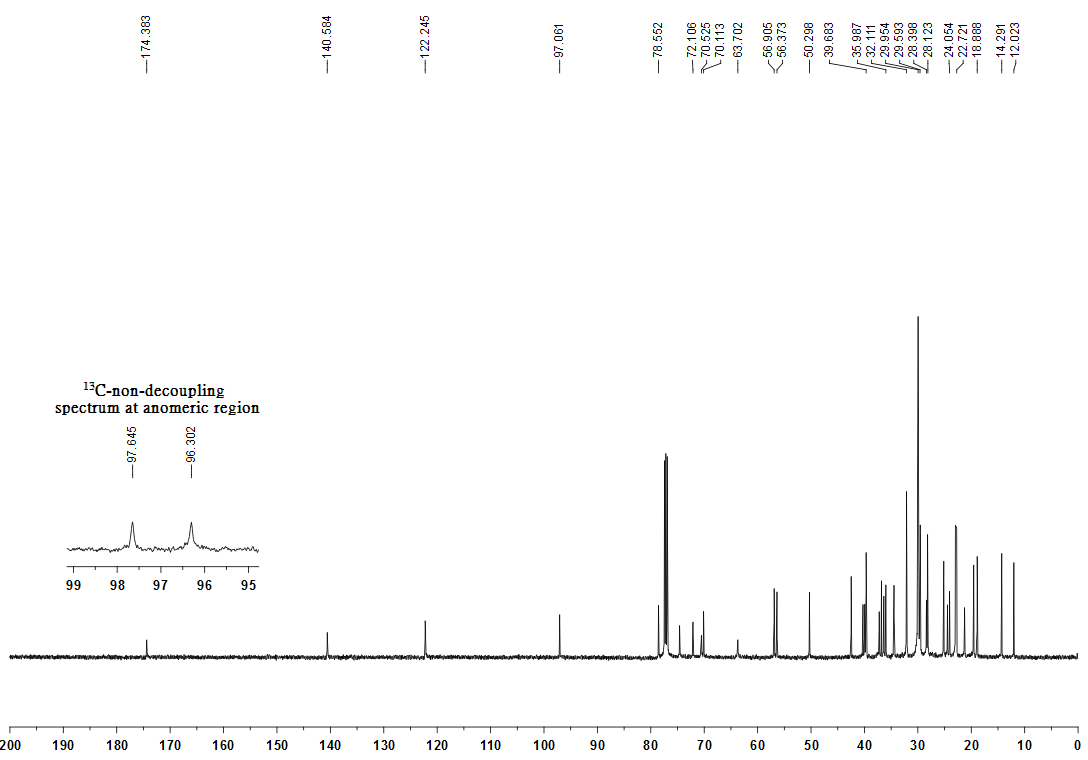


^1^H NMR spectrum of CAG 20:0 (CDCl_3_, 400 MHz)


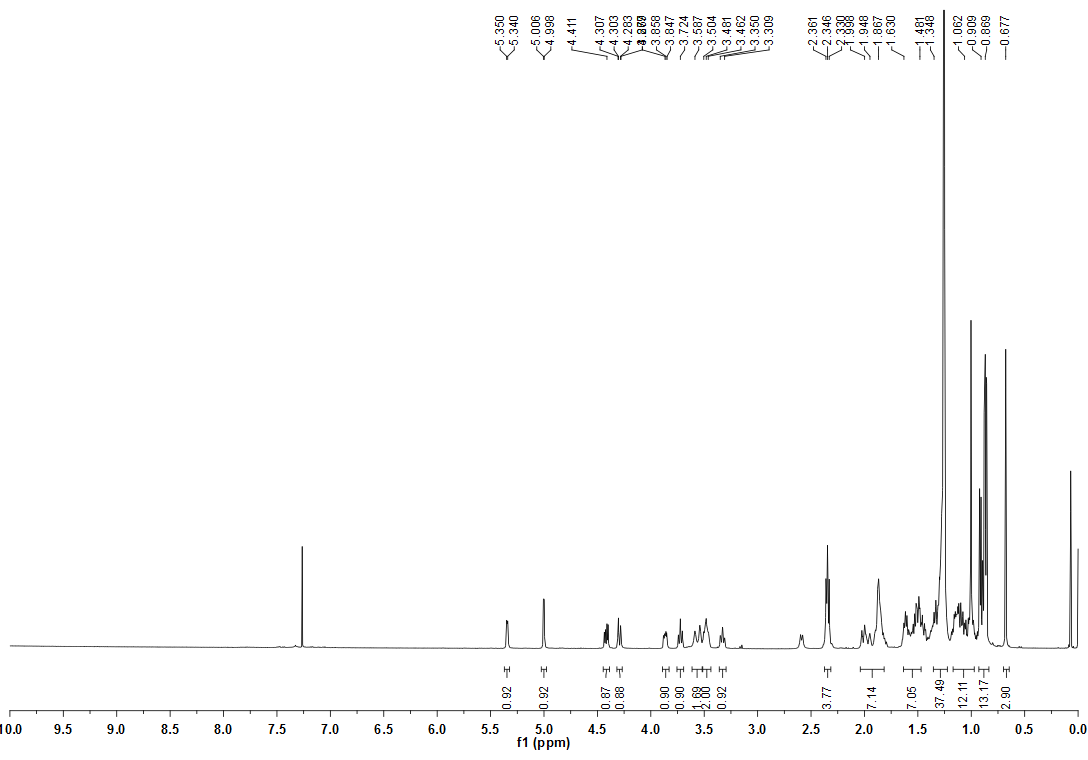


^13^C NMR spectrum of CAG 20:0 (CDCl_3_, 100 MHz)


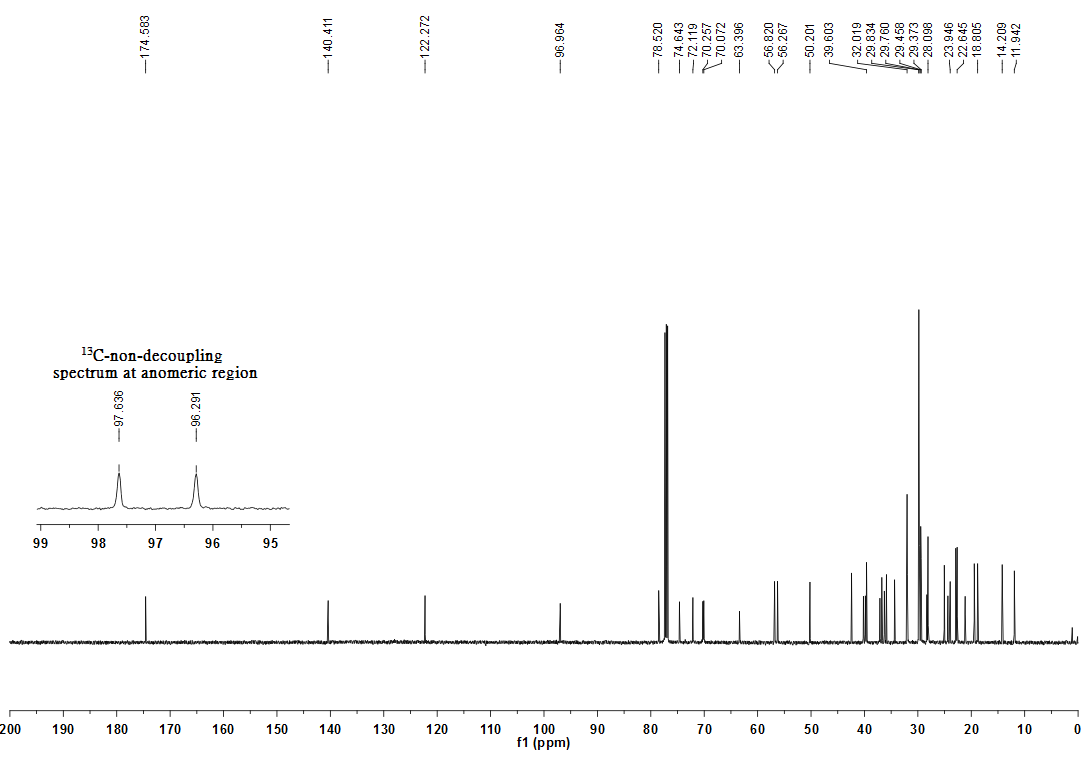


^1^H NMR spectrum of CAG 22:0 (CDCl_3_, 400 MHz)


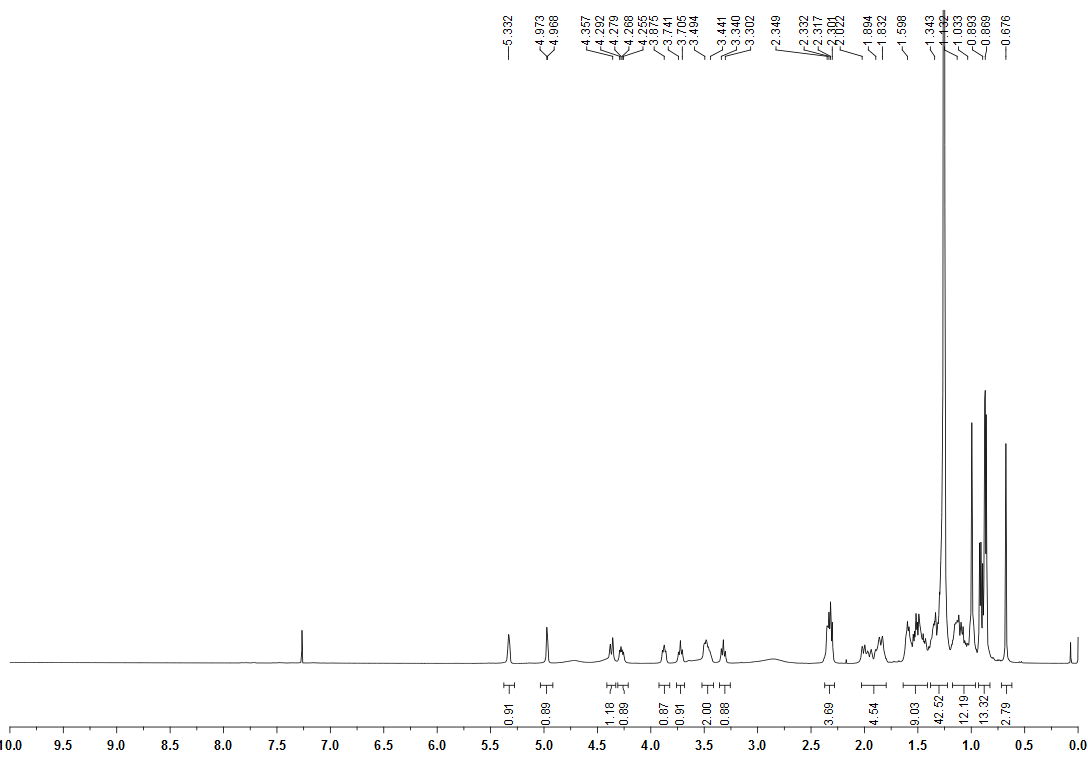


^13^C NMR spectrum of CAG 22:0 (CDCl_3_, 100 MHz)


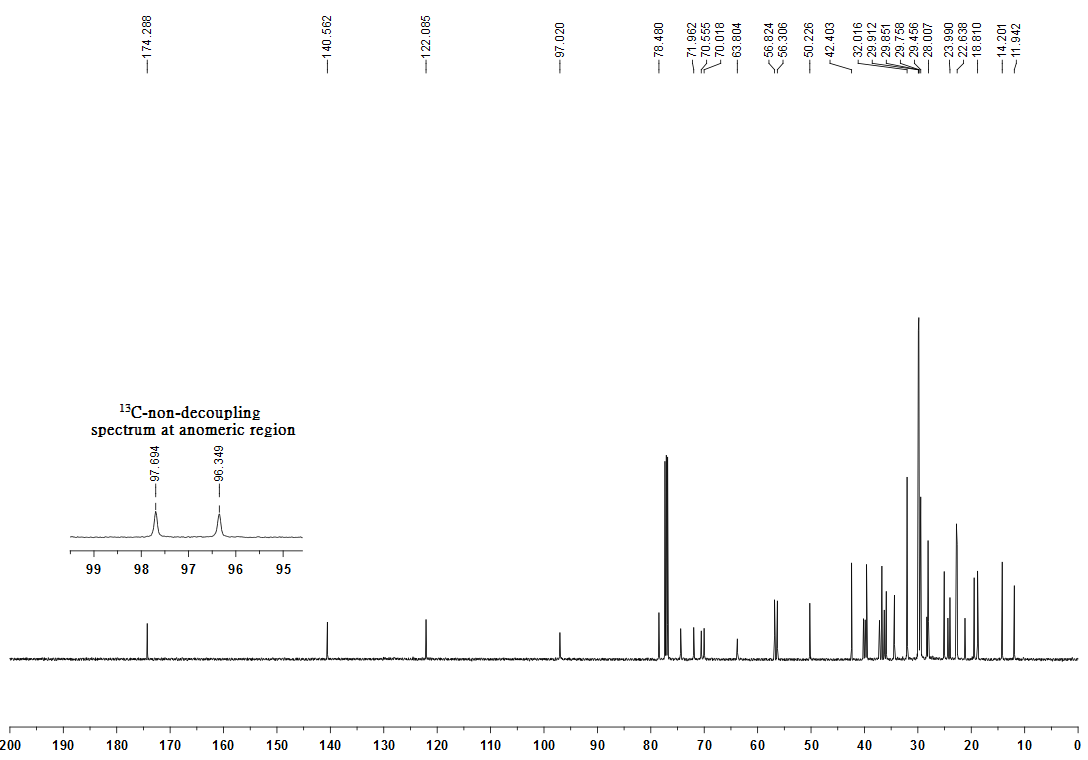


**^1^H NMR spectrum of CAG 18:1 (400 MHz, CDCl_3_)**


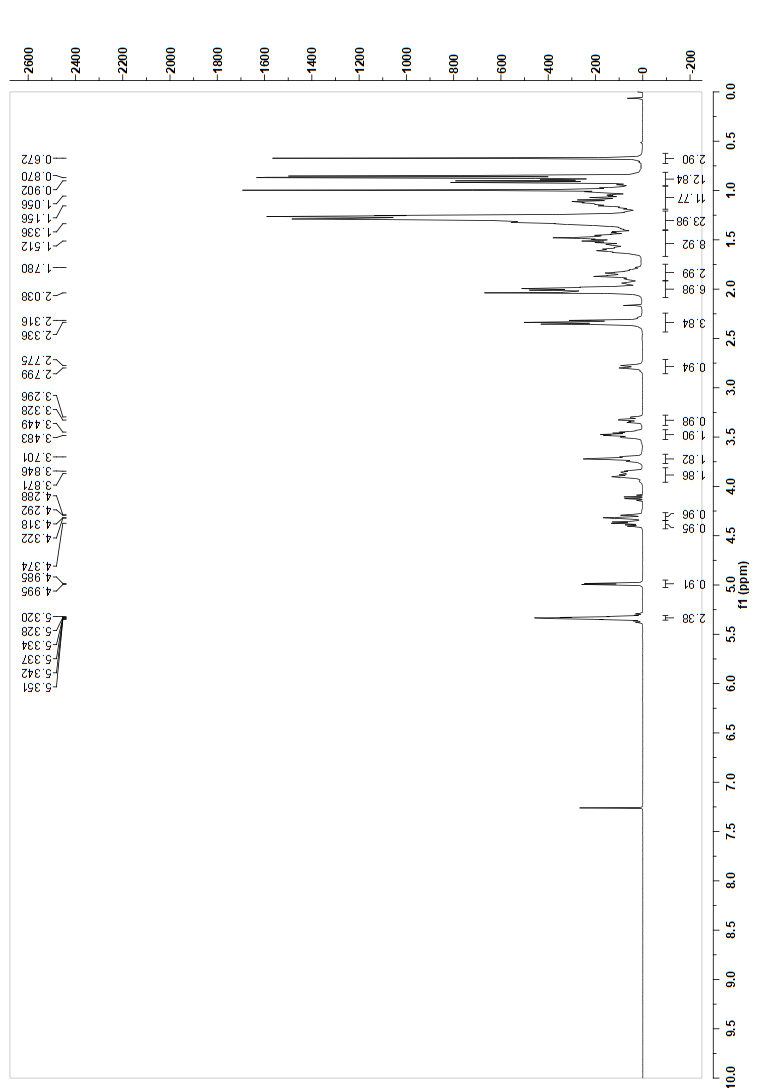


^13^C NMR spectrum of CAG 18:1 (CDCl_3_, 100 MHz)^^


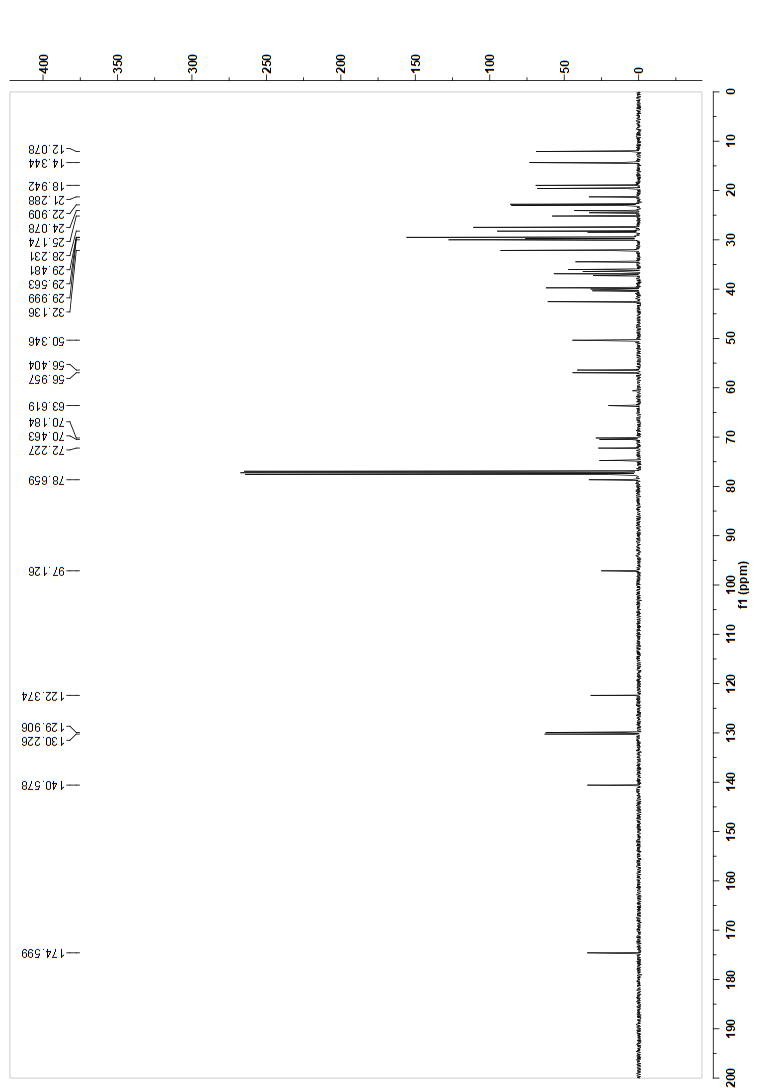


^1^H NMR spectrum of CAG 18:2 (400 MHz, CDCl_3_)


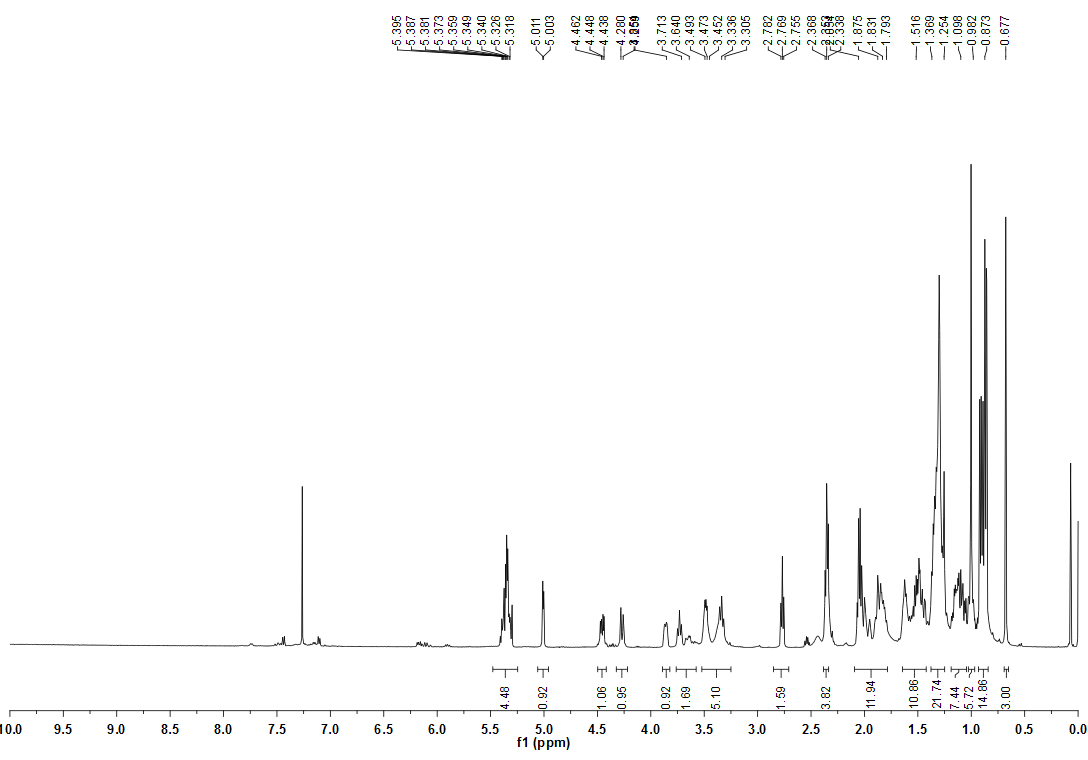


^13^C NMR spectrum of CAG 18:2 (CDCl_3_, 100 MHz)


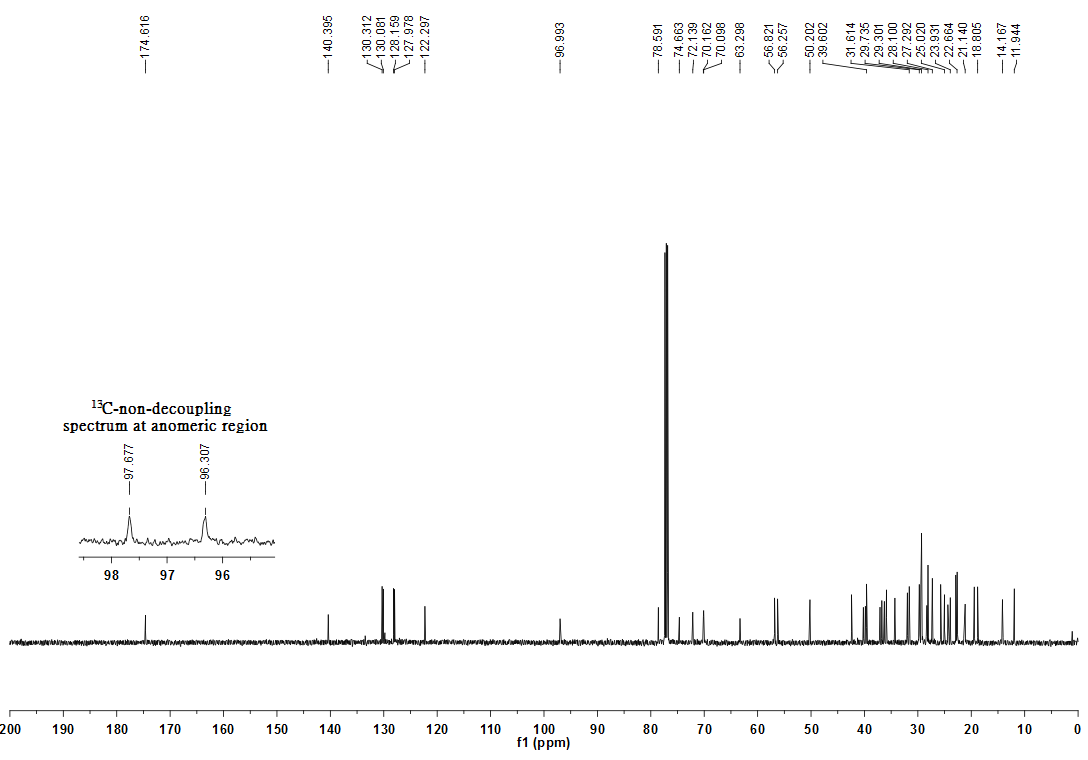


^1^H NMR spectrum of CAG 18:3 (CDCl_3_, 400 MHz)


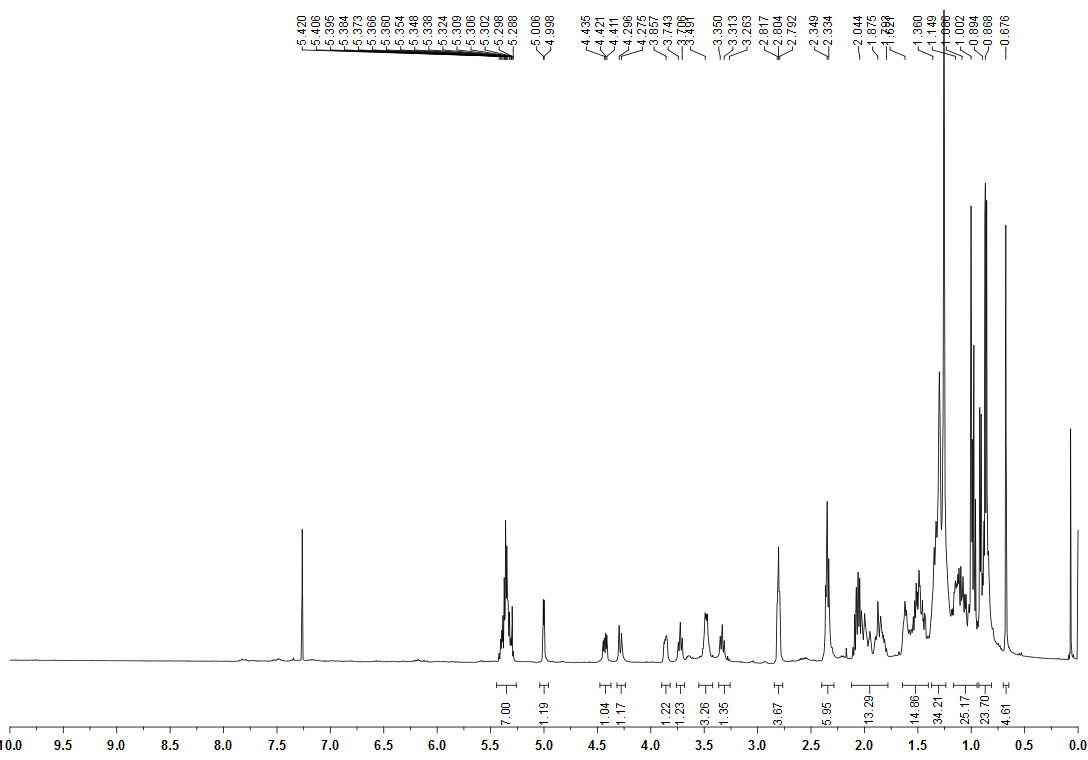


^13^C NMR spectrum of CAG 18:3 (CDCl_3_, 100 MHz)


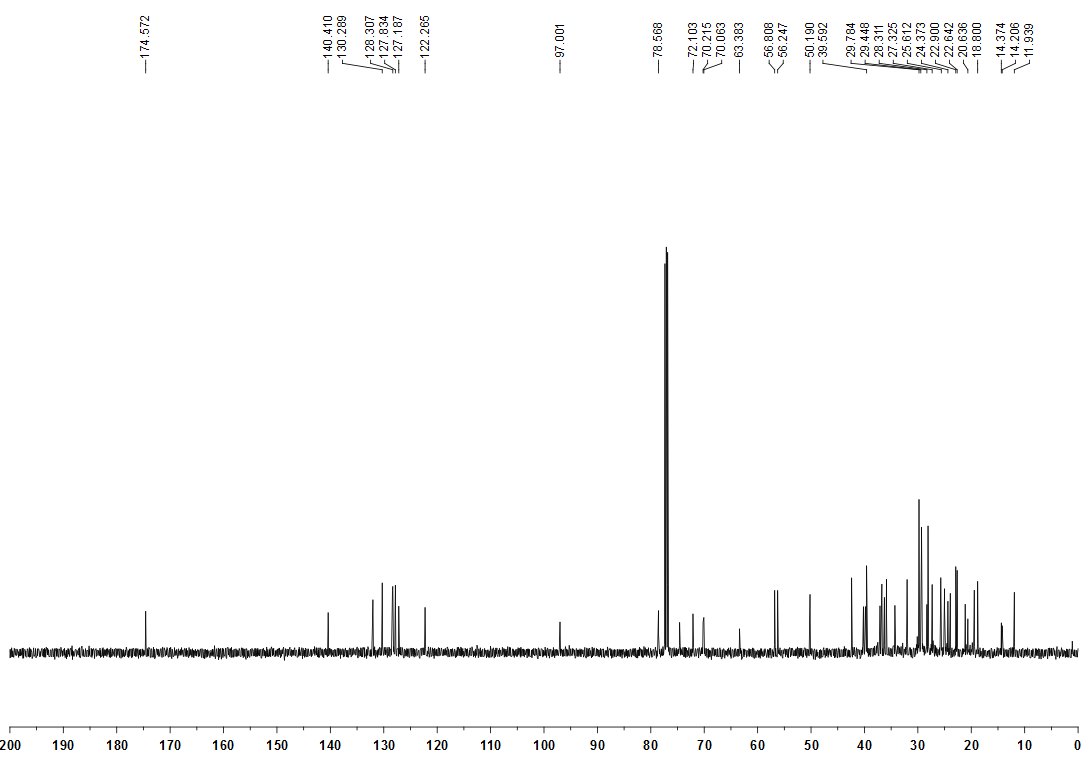


^1^H NMR spectrum of CAG 20:4 (CDCl_3_, 400 MHz)


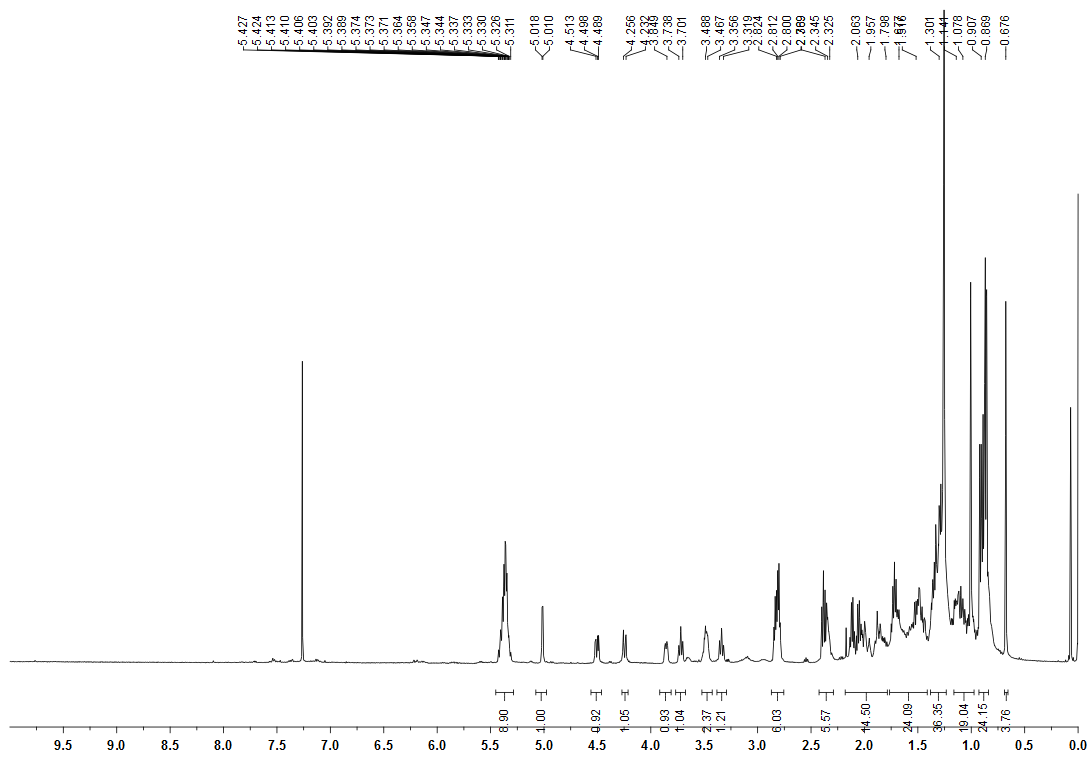


^13^C NMR spectrum of CAG 20:4 (CDCl_3_, 100 MHz)


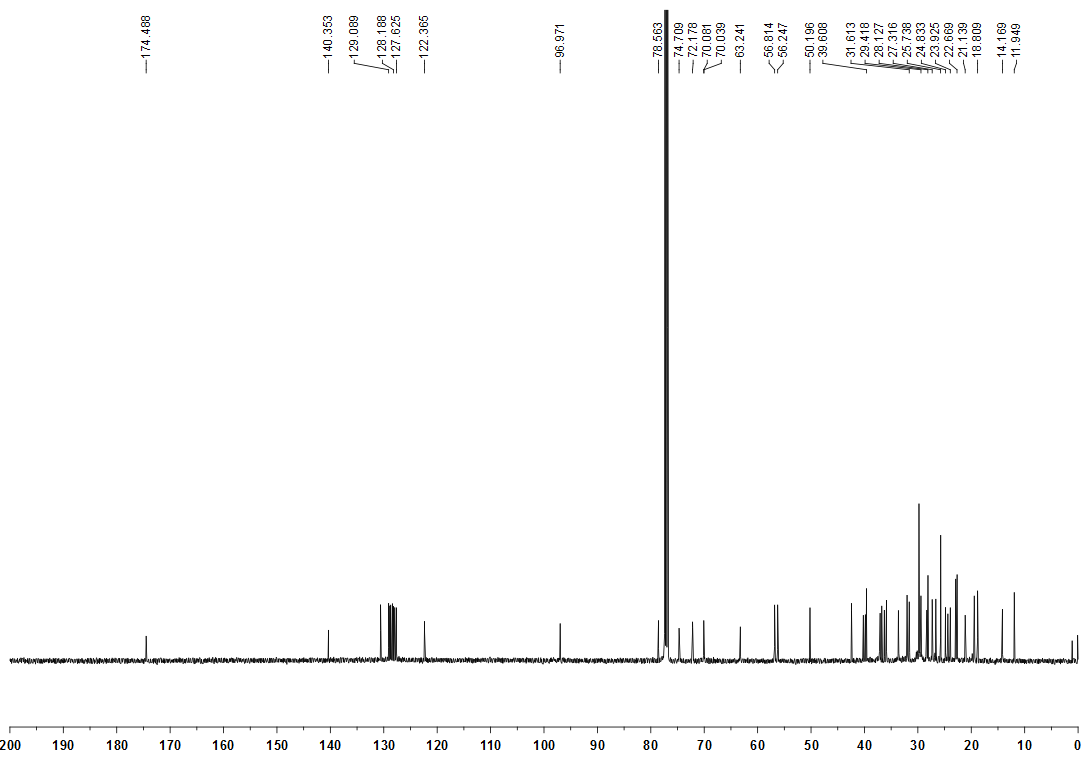


**^1^H NMR spectrum of CAG 22:6 (CDCl_3_, 400 MHz)**

**
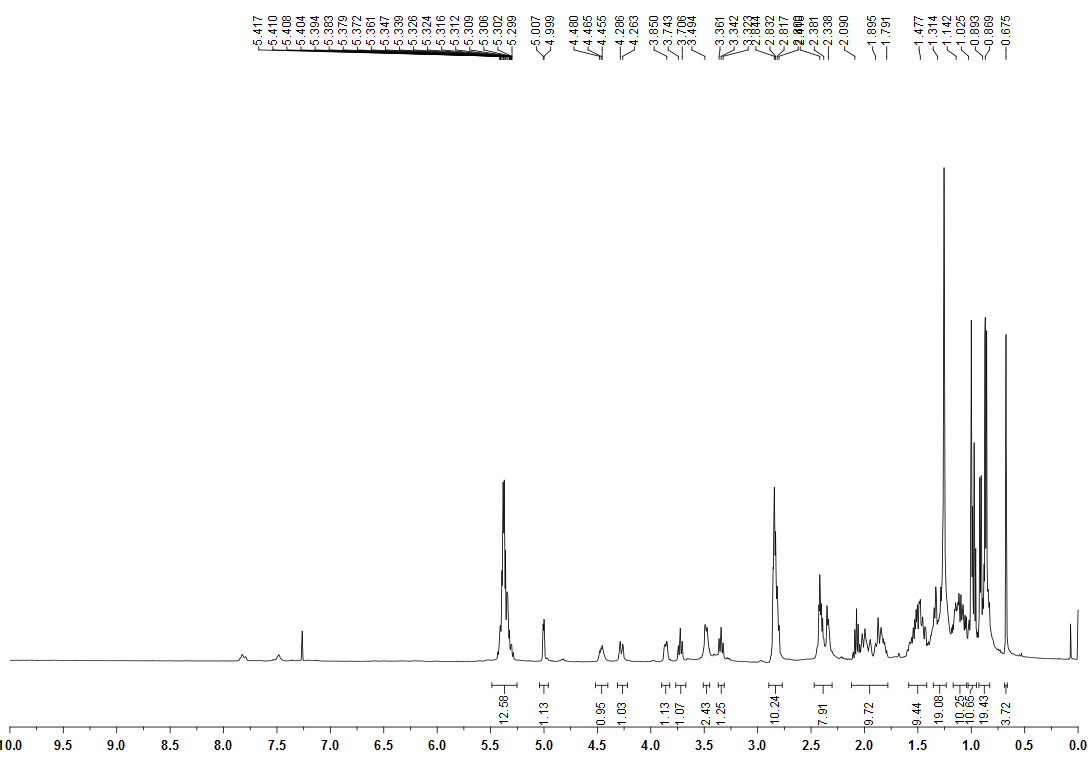
**

**^13^C NMR spectrum of CAG 22:6 (CDCl_3_, 100 MHz)**

**
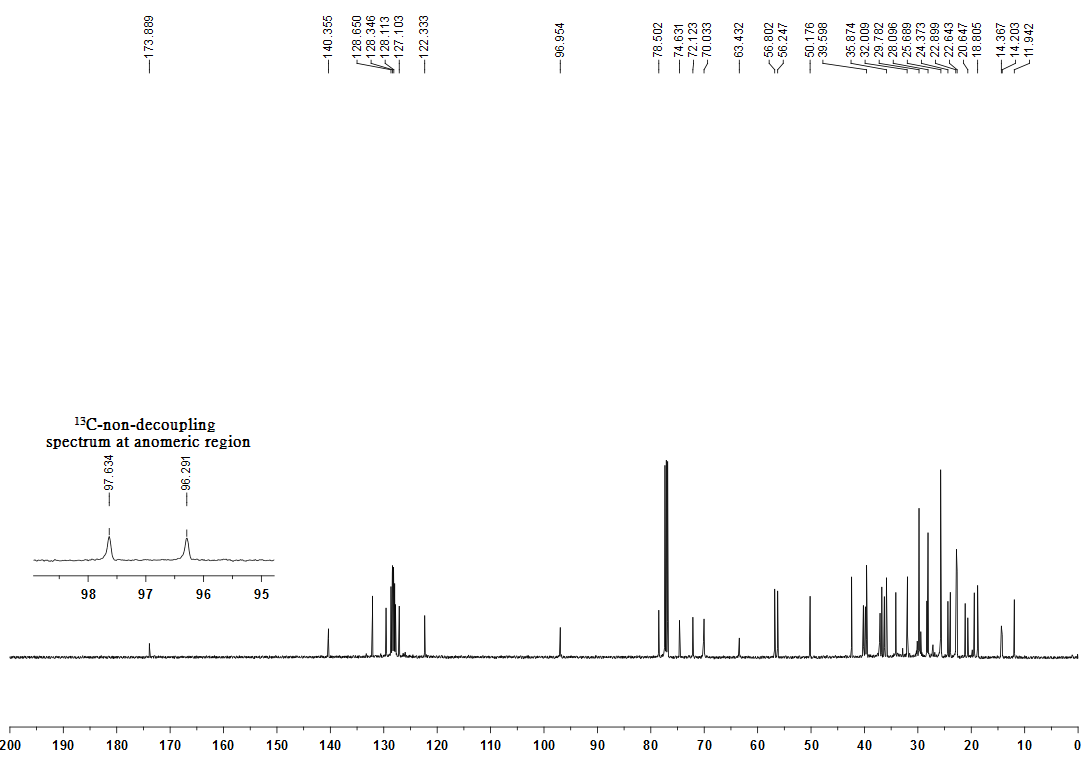
**
